# Supplementary material for: The Molecular Basis of Radial Intercalation during Tissue Spreading in Early Development
Source: Dev Cell. 2016 May 9;37(3):213–25. doi: 10.1016/j.devcel.2016.04.008 (PMC4865533; doi:10.1016/j.devcel.2016.04.008)
Supplement: Document S1. Supplemental Experimental Procedures and Figures S1–S7 [file mmc1.pdf]

Developmental Cell, Volume *37*

## Supplemental Information

# The Molecular Basis of Radial Intercalation during Tissue Spreading in Early Development

**András Szabó, Isidoro Cobo, Sharif Omara, Sophie McLachlan, Ray Keller, and Roberto Mayor**

**Inventory of Supplemental Information**


**Figure S1,** related to Figure 2. C3aR inhibition affects blastopore closure.
**Figure S2**, related to Figure 2. Complement component C3 is required for normal Xenopus epiboly.
**Figure S3**, related to Figure 2. C3aR inhibition does not affect cell-to-cell adhesion.
**Figure S4**, related to Figure 2. Fibronectin and cell-fibronectin adhesion are unaffected by C3aR inhibition.
**Figure S5**, related to Figure 2. Ectoderm and mesoderm specification is not affected by C3aR inhibition.
**Figure S6**, related to Figure 3. Ectoderm and mesoderm specification is not affected by C3aR inhibition.
**Figure S7**, related to Figure 6. Model behavior after changing the number of cell layers.
**Movie S1**. Ex vivo assay of radial intercalation.
**Movie S2**. Computational model simulation of radial intercalation.
**Movie S3**. In vitro chemotaxis assay using deep and superficial layer explants.
**Movie S4**. In vitro chemotaxis assay using deep layer explants and purified C3a.
**Movie S5**. Ex vivo assay showing protrusive activity.
**Movie S6**. In vivo expansion of the blastocoel roof during epiboly.
**Movie S7**. Computational model simulation with altered chemoattractant levels.
**Supplemental Experimental Procedures**
**Supplemental References**

## Supplemental Figures

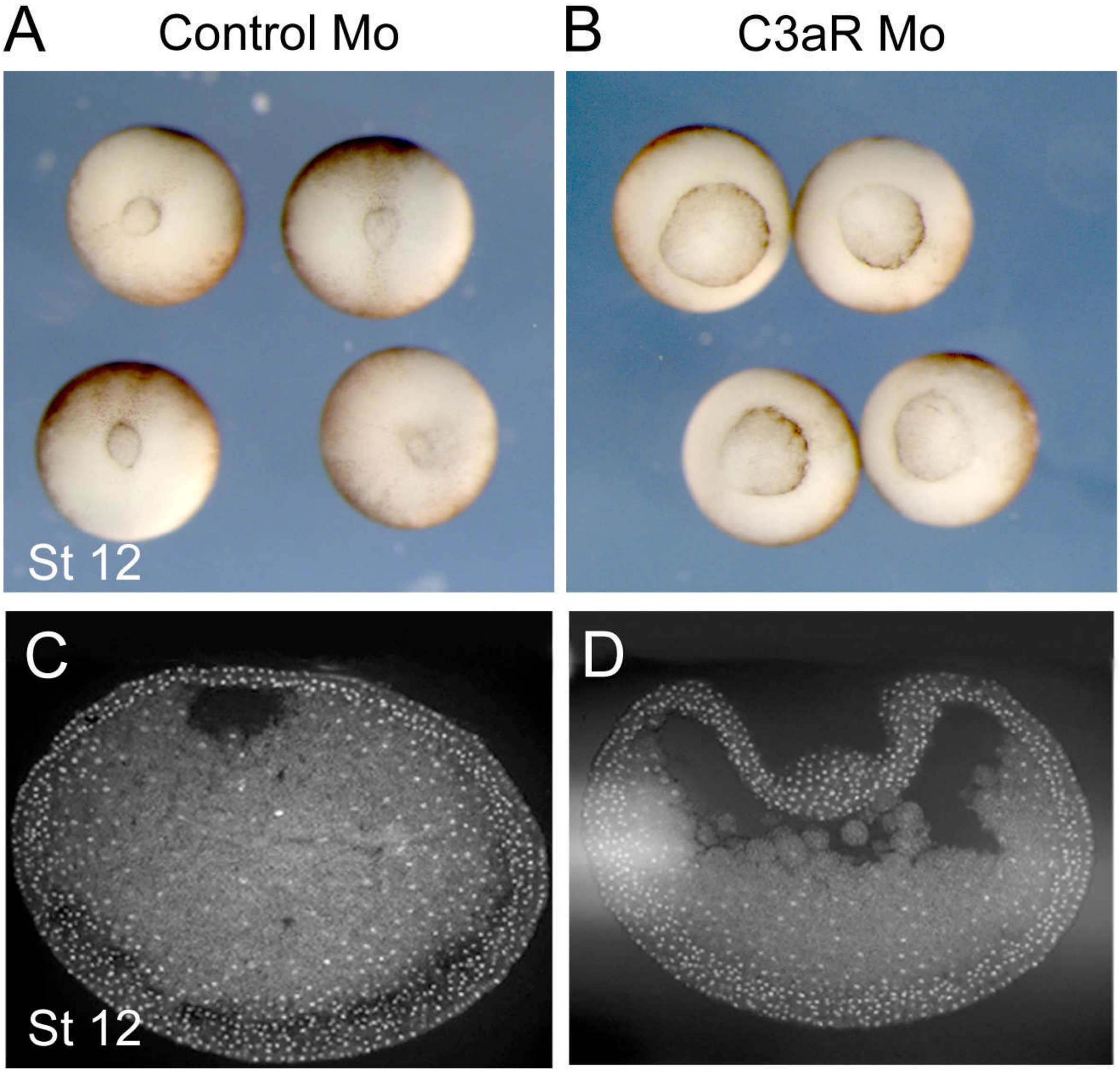


**Figure S1. C3aR inhibition affects blastopore closure. Related to Figure 2**

Blastopore of representative control (A, CoMo injected) and morphant (B, C3aRMo injected) embryos at stage 12. C3aR inhibition leads to failure of blastopore closure in 68% of the observed n=120 embryos. Internal structure of embryos shown on sections of whole control (C, CoMo) and morphant (D, C3aRMo) embryos.

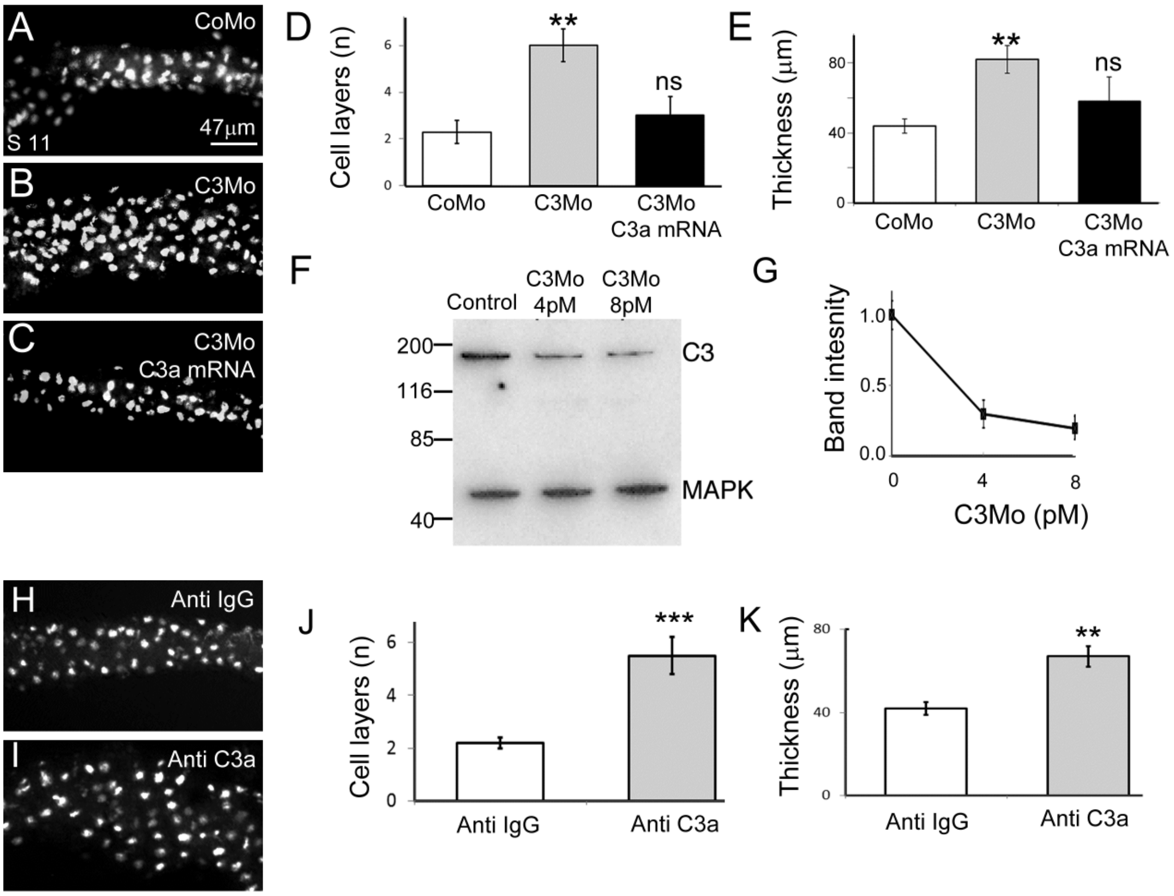


**Figure S2. Complement component C3 is required for normal Xenopus epiboly. Related to Figure 2**

Sections of the BCR of stage 11 embryos treated with CoMo (A), C3Mo (B), and C3Mo co-injected with rescue C3a mRNA that does not hybridize the C3Mo (C). The number of cell layers (D, n=60 embryos, $p<0.01$) and tissue thickness (E, n=60 embryos, $p<0.01$) are both significantly increased in C3 inhibited embryos compared to those in CoMo injected embryos. This effect is rescued by co-injecting C3Mo and C3a mRNA, in which case the difference in number of cell layers and tissue thickness is not significant when compared to those in CoMo injected embryos. This rescue shows the specificity of C3Mo. Western blot of CoMo and C3Mo injected SLs shows the dose-dependent inhibitory effect of the morpholino (F, G). All inhibitory experiments were carried out using 4pM Mo concentrations. (H, I) Sections of BCR of stage 11 embryos injected in the blastocoel cavity with pre-immune anti IgG antibody (H) or a blocking anti C3a antibody (I), previously characterized (Carmona-Fontaine et al., 2011). The number of cell layers (J, n=30 embryos, $p<0.005$) and tissue thickness (K, n=35 embryos, $p<0.01$) are both increased in embryos injected with the C3a antibody, but not with the control IgG. Error bars on D, E, G, J and K represent standard deviation.

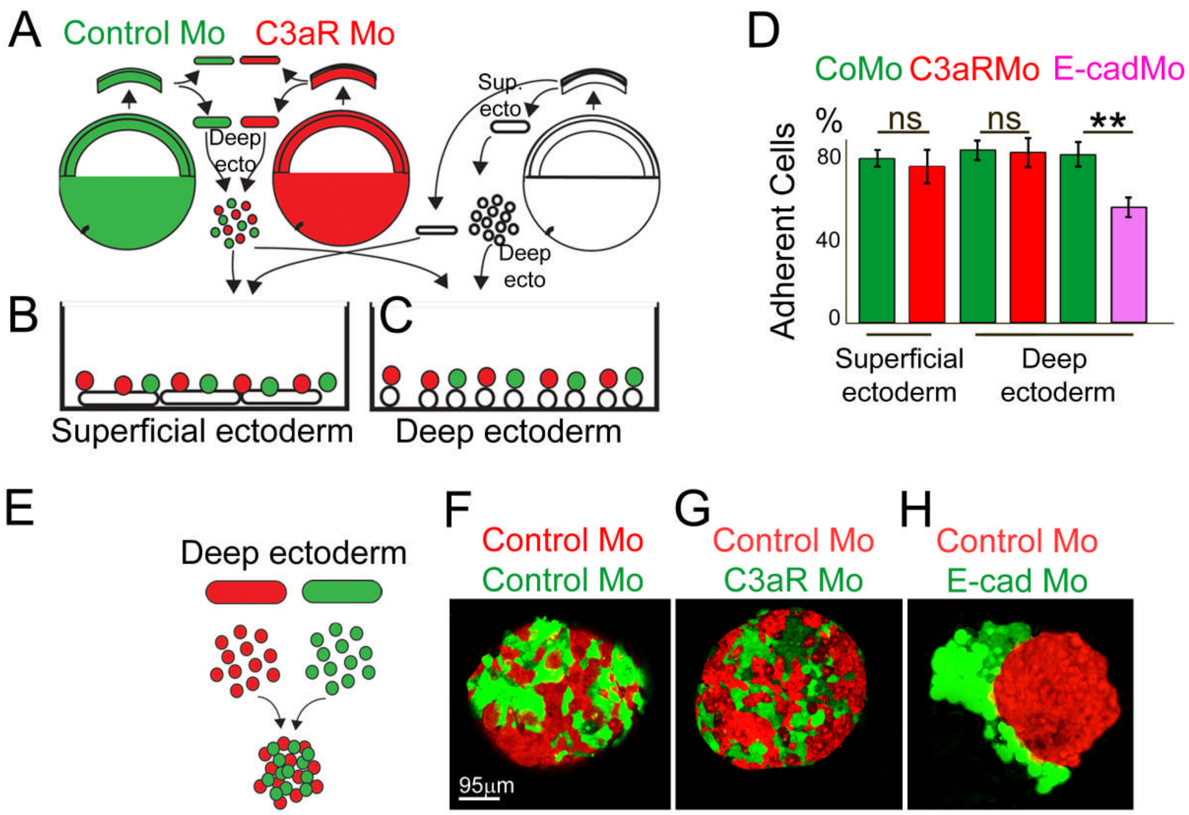


**Figure S3. C3aR inhibition does not affect cell-to-cell adhesion. Related to Figure 2**

(A-C) Deep ectoderm of stage 10 embryos injected with CoMo (green) or C3aRMo (red) were explanted onto a layer of superficial (B) or deep (C) ectoderm of untreated embryos (white, unlabeled). For positive control, deep ectoderm from CoMo and E-Cadherin Mo (E-cadMo) injected embryos were explanted on untreated deep ectoderm (not shown). After 2h the cultures were washed to remove detached cells and the percentage of variously treated cells remaining on superficial or deep ectoderm was quantified (D, n=80 experiments). No significant difference was observed in the attachment of CoMo and C3aR inhibited cells, however, the difference between control and E-Cadherin inhibited cells is significant ($p<0.01$, error bars: standard deviation).

(E-H) Cell sorting assays of deep ectodermal cells from two different stage 9 embryos were performed on agarose covered dishes. No adhesion difference was observed between cells explanted from CoMo and CoMo (F), or CoMo and C3aR inhibited (G) embryos, while cells originated from CoMo and E-Cadherin embryos clearly sorted (H).

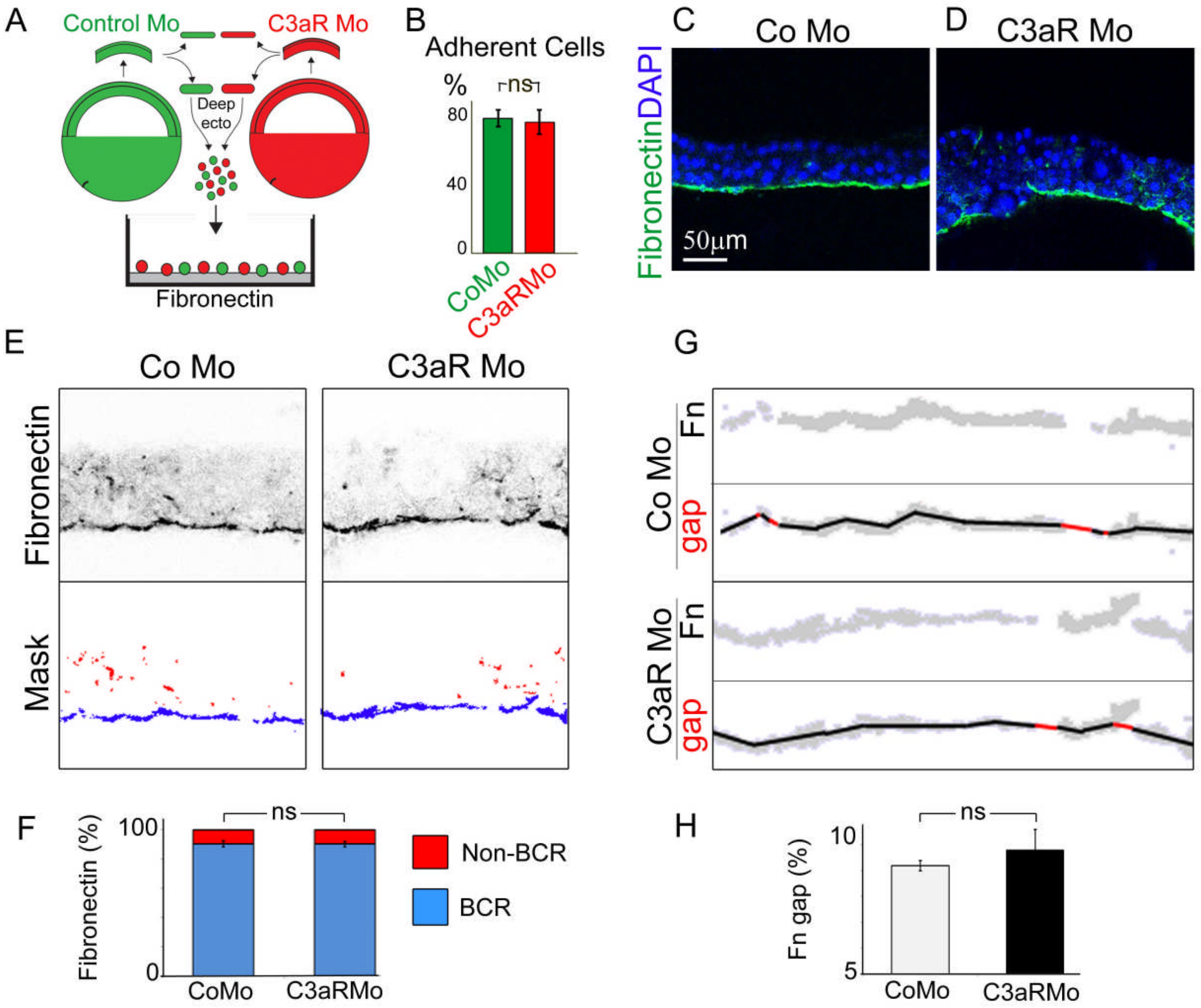


**Figure S4. Fibronectin and cell-fibronectin adhesion are unaffected by C3aR inhibition. Related to Figure 2**

(A-B) Deep ectodermal cells from CoMo (green) and C3aRMo treated (red) embryos at stage 10 were explanted, mixed, and plated on fibronectin substrate. After 2h of culture the cells were washed. (B) The percentage of adherent cells after the wash did not show significant difference between control and C3aR inhibited cells (n=30 experiments, error bars: standard deviation).

(C-H) Fibronectin accumulation at the BCR in vivo is unaffected by C3aR inhibition, as shown by immunostained sections of the BCR from stage 10 embryos (C-D). Most importantly, fibronectin is accumulated at the edge of the BCR despite the heavily disrupted tissue architecture. Note the occasional slight ectopic expression and discontinuity in the fibronectin in both CoMo (C, middle and right side) and C3aRMo (D) treated embryos.

Quantifications of fibronectin immunofluorescence intensities at the blastocoel roof (BCR, blue) and within the tissue (Non-BCR, red) show no significant difference between the control (CoMo) or morphant (C3aRMo) embryos (F). Continuity of the fibronectin layer at the BCR was assessed by measuring the length of BCR segments without fibronectin (gaps; G, red). This is then related to the total length of the BCR (red and black lines). No significant difference was found between control (CoMo) and treated (C3aRMo) embryos (H). Error bars: standard error of the mean.

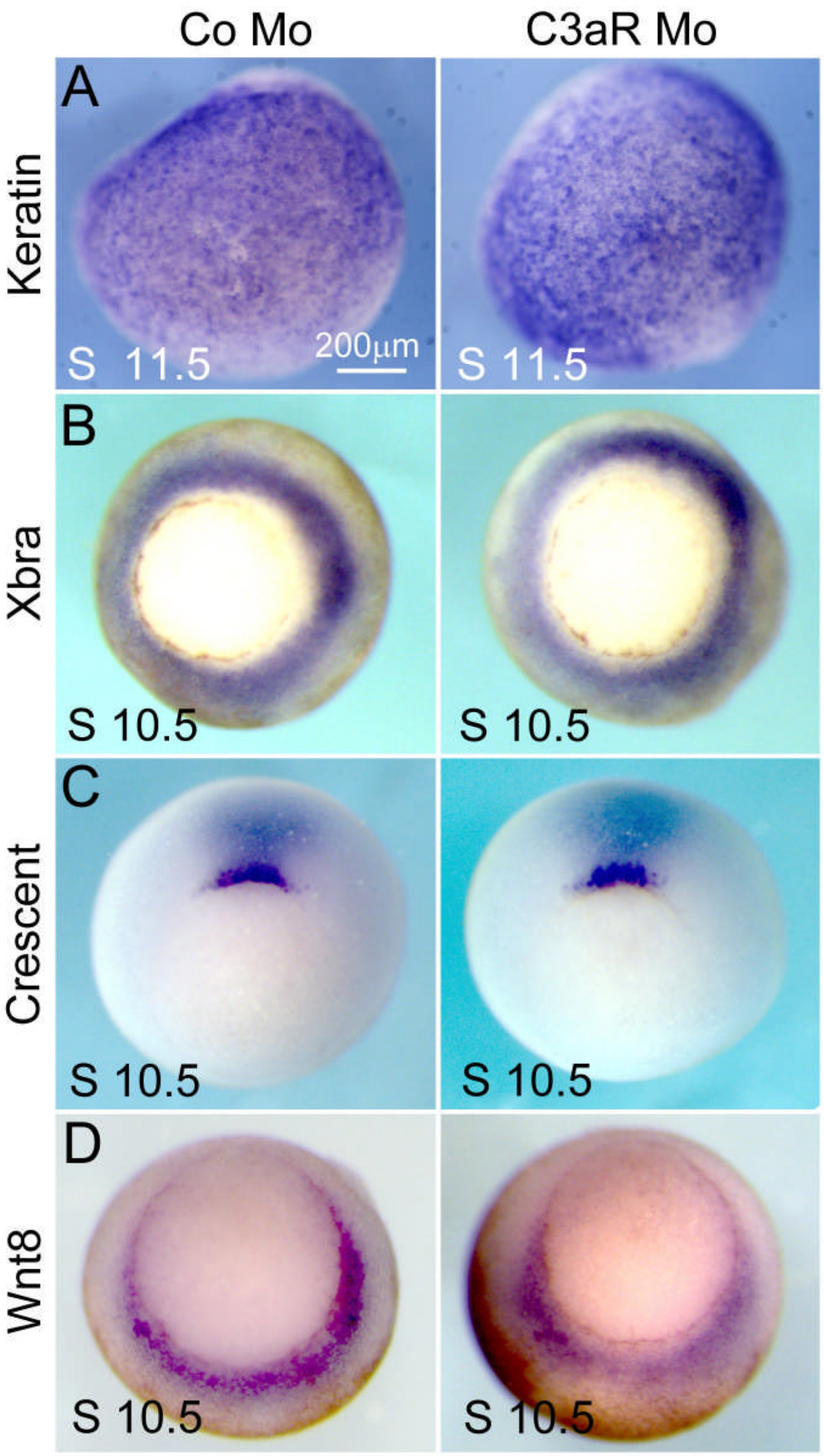


**Figure S5. Ectoderm and mesoderm specification is not affected by C3aR inhibition. Related to Figure 2**

(A) Keratin expression in the animal pole at stage 11.5 is normal in C3aRMo injected embryos, indicating the normal specification of the ectoderm (ISH, animal view).

(B-D) Mesodermal markers Brachyury (Xbra), Crescent, and Wnt8 at stage 10.5 are unaffected by C3aR inhibition (ISH, vegetal view).

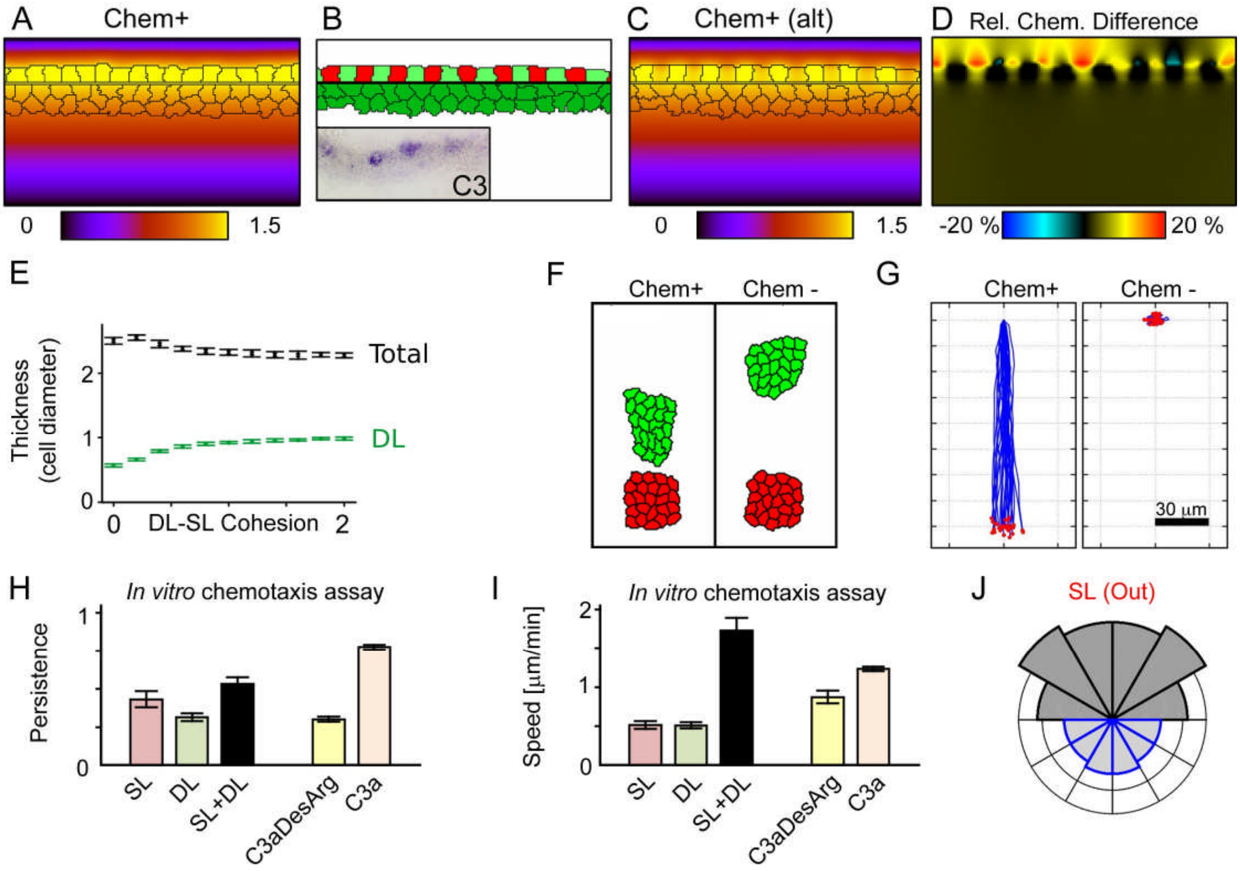


## Figure S6. Computational model behavior. Related to Figure 3

(A-D) Alternating chemoattractant expression does not affect the chemoattractant gradient in the simulations. (A) Emergent chemoattractant field with overlaid cell outlines in a simulation where all SCs secrete. (B) Configurations using alternative chemoattractant expression inspired by the patterned ISH signal observed in animal pole sections (inset). (C) Chemoattractant field with overlaid cell outlines in the simulation shown in (B). (D) The point-by-point relative difference of the uniform ($c_u$, shown on A) and alternating ($c_a$, shown on C) chemoattractant fields defined as: $(c_u\text{-}c_a)/c_u$, diminishes further away from the SL where the difference is less than 10% homogeneously.

(E) Thickness of the whole BCR tissue (Total) and the DL as the function of effective friction between the superficial and deep layers (DL-SL Cohesion). Without cohesion the DL thins out, and as the friction is increased, DL thickness reaches a plateau within less than double of the minimal value. Meanwhile the total tissue thickness is largely unaffected. All simulations in the study are run with cohesion parameter = 1, the smallest value where the DL thickness starts to reach the plateau. Error bars represent standard deviation, n=30 simulations for each parameter set.

(F) Chemotaxis assay in silico showing chemotaxis of a DL explant (green) towards a cluster of SL explant (red) with (Chem+) and without (Chem-) chemotaxis. Explants are simulated on a 2D substrate.

(G) Trajectories of DL explants in silico with and without chemotaxis.

(H-I) Persistence (H) and speed (I) of clusters in the in vitro chemotaxis assay shown in Figure 4. Error bars: standard error of the mean.

(J) Distribution of protrusion directions of in vivo DCs that are completely surrounded by other cells, quantified from SEM images of fixed and fractured embryos (see main Figures 5A-D). This measurement excludes any potential bias towards the SL due to the lack of cell neighbors towards the blastocoel at the very bottom of the DL. This restricted distribution still shows a significant bias towards the SL, similar to Figure 5E.

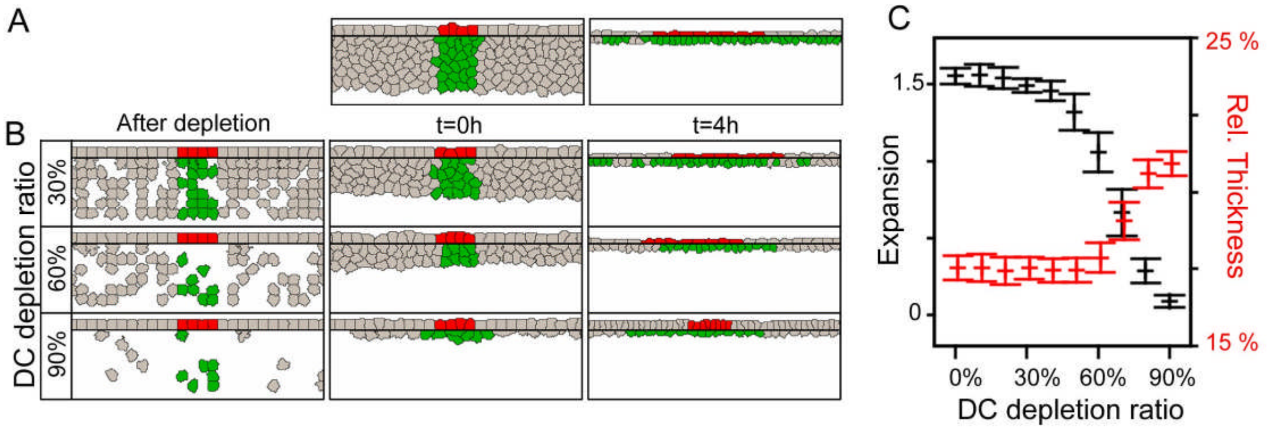


**Figure S7. Model behavior after changing the number of cell layers. Related to Figure 6**

(A) Configurations from a sample simulation run with 6 layers of DCs showing that the model reproduces epiboly in systems with more than 2 layers of DCs, as in zebrafish.

(B) Simulations initiated with 6 layers of DCs, after which a given percentage of the DC population (DC depletion ratio) is removed. The resultant holes are filled by the DCs condensing in the vicinity of the SL. The proposed chemotaxis-based mechanism in simulations with a large number of DCs produces effective expansion of the tissue. Expansion is hampered only when the number of DC layers falls below two.

(C) SL expansion and relative tissue thickness in simulations run with different ratio of DCs depleted from the initial 6 layers of DCs (see panels I and J). Thickness is relative to the initial tissue thickness (before depletion). Even when the majority of the DCs are depleted from the simulation, expansion is still observed due to the local expansion transmitted to the SL. N=30 independent simulations per condition, error: standard deviation.

## Supplemental Movie captions

### Movie S1. Related to Figure 2, 3 and 6

Deep layer intercalation and expansion depends on C3aR, as shown by time-lapse microscopy using the ex vivo assay (see Figure 2D). Mosaic-labeled deep layers (membrane – green, nuclei – red) explanted and covered with an unlabeled superficial layer were imaged through the superficial layer. Manually tracked cell trajectories demonstrate expansion (magenta tracks) in the control (CoMo treated deep layer) case (top row), but not in the C3aRMo case. Deep cells from lower layers intercalate into the top layer (cyan tracks), whereas cells from the top layer disappear when moving into deeper layers (blue track). Note that images of both CoMo and C3aRMo explants contain the same number of cells and the dark areas show unlabeled DCs. Any apparent difference in number of cells is due to natural variation in the mosaic labeling. Right column shows the trajectories without the fluorescent images. Framerate 3 minutes per frame.

### Movie S2. Related to Figure 3

Cellular Potts model simulation of chemotaxis-driven epiboly. Two independent simulations demonstrate radial intercalation and expansion with chemotaxis (top, Chem+) and the lack of epiboly in the inhibited case (bottom, Chem-). Blue bars mark the initial position of the colored region (superficial cells – red, deep cells – green). Cell coloring is for visualization purposes only. Framerate 3 minute per frame (1800 MCS per frame).

### Movie S3. Related to Figure 4

In vitro chemotaxis assay of deep and superficial layer explants. Deep and superficial layers explants are not motile when plated separately. When plated in proximity, control (CoMo) deep layer explants chemotax towards CoMo superficial layer explants. Chemotaxis is lost in experiments where the superficial layer is explanted from a C3Mo injected embryo, or when the deep layer is explanted from a C3aRMo injected embryo. Bottom row shows trajectories of 10 explants. Framerate 3 minutes per frame.

### Movie S4. Related to Figure 4

Deep layer explants chemotax towards C3a source. Inverted bright-field time-lapse recordings of deep cell explants plated near the vicinity of C3a-soaked beads (circled). Explants do not chemotax towards beads soaked in C3aDesArg (left), a variant of C3a that is unable to bind to its receptor (C3aR). Bottom row shows trajectories of 10 explants. Framerate 3 minutes per frame.

### Movie S5. Related to Figure 4

Apical protrusions (A, B) and protrusive activity (C, D) in deep layers covered with superficial layer in our ex vivo assay. Mosaic-labeled deep cells explanted and covered with an unlabeled superficial layer were imaged through the superficial layer. Control (CoMo) deep cells produce protrusions (A) and exhibit more protrusive activity (C, pseudo-colored to yellow) than C3aRMo treated deep cells. Framerate 3 minutes per frame.

### Movie S6. Related to Figure 6

In vivo expansion of the blastocoel roof is dependent on C3aR. Animal pole of Xenopus embryos imaged from stage 8 show expansion in control (CoMo injected) case. This expansion is reduced when C3aR is inhibited (C3aRMo). Left column shows fluorescent time-lapse (membrane – green, nuclei – red) movie with manual trajectory overlay. Right column shows the trajectories only. Framerate 10 minutes per frame.

### Movie S7. Related to Figure 7

Model simulation of epiboly at altered chemoattractant levels. Two independent simulations showing epiboly with 0.5x (top) or 1.5x (bottom) levels of chemoattractant compared to simulations shown in Movie S2. Both expansion and thinning is reduced when chemoattractant levels are halved, and increased with increased chemoattractant levels. Colors and marking as in Movie S2. Framerate 3 minute per frame (1800 MCS per frame).

# Supplemental Experimental Procedures

## Morpholinos and peptides

Xenopus C3a and C3aDesArg peptides were synthesized as previously described (Carmona-Fontaine et al., 2011). The following Morpholino oligomers were designed according to recommendations of Gene Tools or using published data:

- C3Mo: 5'-ACTGGACAATGTGCAAACTTTGAAT-3';
- C3aRMo and CoMo: as published (Carmona-Fontaine et al., 2011);
- E-cadherin Mo: as published (Nandadasa et al., 2009).

The effectiveness of the C3Mo is demonstrated in Figure S2F and S2G using western blot. The fibronectin antibody used was the mAb 4H2 previously characterized in the Xenopus system (Ramos and DeSimone, 1996).

## Staining and Imaging

Whole-mount in situ hybridization was performed as described previously (Harland, 1991).
For in vivo time-lapse imaging of the SL, the animal pole of embryos was co-injected with nuclear RFP and membrane GFP constructs at the 8-cell stage, together with either CoMo or C3aRMo constructs. Embryos were imaged from the animal pole using fluorescence confocal microscopy (Leica, Upright-SP8) with several optical sections that were later max-projected using ImageJ. In vitro imaging was carried out using standard time-lapse fluorescence microscopy. Images were captured every 3 or 10 minutes as indicated.
Automatic tracking of cell nuclei of in vivo SL (Figure 6E and 6F) was performed using the DCU CIPA TRACKER software (Hossain et al., 2011; Thirusittampalam et al., 2013). Manual tracking was done using ImageJ's Manual Tracker plugin.
Adhesion and sorting assays were performed as previously described (Carmona-Fontaine et al., 2011).

## Chemotaxis assay

For in vitro chemotaxis deep and superficial layers of the BCR were excised from embryos between stages 10 to 12 and separated by shaving the deep layer from the superficial in 3/8 NAM. Explants were plated on FN-coated (10µg/mL, Sigma) plastic dishes filled with DFA (Keller, 1978). Chemotaxis assays were performed and analyzed as described previously (Carmona-Fontaine et al., 2011). Briefly, explants of deep and superficial layers from stage 10 embryos were placed either separately, or at a distance of approximately one explant width. A similar procedure was carried out for C3a-coated and C3aDesArg-coated beads. A minimum of 10 explants were imaged for at least 6h for each condition. Chemotaxis indices were calculated using the Chemotaxis and Migration Tool for ImageJ (Ibidi).

## Ex vivo tissue explant

Ex vivo intercalation assays were performed as previously described (Marsden and DeSimone, 2011). Briefly, embryos were injected to label the membranes and nuclei of cells in a mosaic fashion. The DL was explanted as described above and covered with SL from unlabeled embryos. The whole explant was covered with a glass cover slip to prevent curling of the SL. The optical penetration depth was limited to the top DC layer, allowing the study of apical DC activity and quenching all signals from the deeper regions. Membrane protrusion activity was determined by thresholding the level of membrane GFP staining signal. A higher signal indicates more membrane overlap caused by the wrinkling, or ruffling, of the cell membranes. The threshold was set to levels corresponding to a smooth cell membrane. Areas with a signal above the threshold were considered as areas of protrusive activity. Intercalation percentages for Figure 2F were calculated as the number of fluorescent cells appearing / disappearing over the time-lapse recordings in relation to the total number of fluorescent cells in the last frame of the recordings.

## Computer simulations

To model our hypothesis we chose the cellular Potts model (CPM) framework (Graner and Glazier, 1992; Glazier and Graner, 1993; Szabó and Merks, 2013), a stochastic Monte-Carlo based model system. Cells are represented as connected domains on a grid with an integer, $\sigma(x)$, denoting the cell id at every grid point $x$. Cell-free areas are designated with a zero id. The same grid is used to describe the concentration field with $c(x)$ at every point $x$. Each configuration $\xi$ is characterized with a scalar valued function, the Hamiltonian:

$$H(\xi) = \sum_{i=1}^{N} \lambda_V (V(i) - V_T(i))^2 + \sum_{<x,y>} J_{x,y}\big(1 - \delta(x,y)\big) \tag{1}$$

The first sum runs through the cells and contains the deviation of cell volume $V(i)$ from a designated target volume $V_T(i)$ scaled with a model parameter $\lambda_V$. The second term sums cell boundaries with weights $J_{x,y}$ corresponding to boundaries between cell types $x$ and $y$. The δ(x,y) is the Kronecher-delta function: $\delta(x,y)=1$ if $x=y$, otherwise $\delta(x,y)=0$. Cell movement is the result of a series of attempts to update cell ids on the grid. In an attempt a grid point, $a$, and one of its neighbors, $b$, are selected at random, and the id from site $b$, $\sigma(b)$, is copied into site $a$. This attempt is then evaluated and accepted with a probability:

$$P(\sigma(b) \to a) = \min(1, \exp\{w(\sigma(b) \to a) - \Delta H(\sigma(b) \to a)\}). \tag{2}$$

The function $\Delta H(\sigma(b) \to a)$ is the change in the Hamiltonian resulting from the copy attempt, while $\Delta w(\sigma(b) \to a)$ is defined as:

$$w(\sigma(b) \to a) = \lambda_c\big(C(b) - C(a)\big) + \sum_{i=\{\sigma(a),\sigma(b)\}} \Delta r_i \cdot \sum_{j\epsilon\{n(i)\}} \lambda_{i,j} \frac{r_{i,j}}{|r_{i,j}|} \cdot \big(|r_{i,j}| - d_{i,j}\big), \tag{3}$$

with $C(a)$ describing the saturated concentration at point $a$ with saturation parameter $s$ as

$$C(a) = \frac{c(a)}{s \cdot c(a) + 1}. \tag{4}$$

These rules give rise to cells with a volume fluctuating around a given value (volume term, Eq. 1), a surface tension-like cell adhesion (adhesion term, Eq. 2). Chemotaxis is implemented in the usual way by biasing cell displacements towards higher concentrations of the $c(x)$ field (chemotaxis term, Eqs. 3 and 4). Cells are allowed to attach to one another upon certain conditions through a conditional attachment term (elastic term, Eq. 3) with $\Delta r_i$ denoting the displacement of cell $i$'s center of mass, $n_i$ the connected neighbors of $i$, $r_{i,j}$ the vectorial distance of cells $i$ and $j$, and $d_{i,j}$ the target distance of connected cells $i$ and $j$. This elastic term allows the construction of the epithelium and adhesion between the deep and superficial layers by forming elastic connections between cells as described below. The unit of time in the simulations is the Monte Carlo step (MCS) which is N copy attempts where N is the number of grid points in the system.

The chemoattractant, C3a, is diffusing and decaying with a constant rate:

$$\partial_t c(t,x) = D\nabla^2 c(t,x) - d_c \cdot c(t,x) \tag{5}$$

and is produced by the cells such that $c(x)=c_0$ = constant for every point $x$ within the secreting cells. Concentrations at the top and bottom boundaries are set to zero, as any chemoattractant is expected to be diluted infinitely within the blastocoel cavity or outside the embryo. Concentrations at the simulation's side

boundaries are constructed to prevent gradients forming along the layers horizontally. This is achieved by copying the concentration values at 30 sites from the side edge to all the sites up to the edge:

$$c_L(x) = c_L(i,j) = c(A,j) \tag{6}$$

for the left edge of the simulation, where *A=30* demarks the outside margin in the simulation. Similarly on the right edge:

$$c_R(x) = c_R(i,j) = c(W - A,j) \tag{7}$$

where *W=400* is the width of the simulation field. In both cases *j* runs through the complete height, 80 lattice sites, of the simulation area.

Cell configurations in the simulations represent a single-cell thick quasi-2D cross sectional slice of the blastocoel roof, including two layers of deep cells (DCs) and a single layer of superficial cells (SCs, see Figure 3B). In simulations exploring the effect of DC numbers on epiboly (Figure S7), simulations are initiated with 6 layers of DCs and a given percentage of the DC population is then selected and removed at random. The SL and DL are separated by a thin and rigid layer of “frozen cells” (“wall cells” or WCs) that serve to prevent DC intercalation into the superficial layer (SL), and to prohibit DCs from exerting unrealistic pressure on the SL. Since cell movement in the model is relative to the simulation grid, DCs could easily push the SL further out as if they were plated on a 2D substrate. However, in the embryo no such substrate exists as the cells adhere only to each other, and such a pressure would not be feasible. WCs are minimal in size (a single lattice site), and are not allowed to move, and form the wall layer (WL).

The epithelium is constructed as a chain of cells attached to the WCs. If the contact area of a SC and the WL is less than a threshold of 2 lattice sites, the SC is forced towards the WL by applying a constant force on the center of mass of the SC. Similarly, if the contact area of two neighboring SCs falls below a threshold of 2 units, the two cells are forced to move towards each other by the means of the elastic constraint (Eq. 3). Otherwise the links among SCs remain inert (by setting $\lambda_{i,j} = 0$). Each SC is connected to exactly two other SC neighbors via such “epithelial” connections. To allow full epithelial integrity, SCs are also required to remain simply connected (via the Global Connectivity plugin; Swat et al., 2012). This construction of the epithelium allows the layer to adjust its height and length without enforcing a fixed cell-cell distance, but still keeps the integrity of an epithelium.

DCs undergo chemotaxis towards the chemoattractant (Eq. 3) and therefore towards the SL. When in contact with the wall cell layer (WL), an elastic link between the cell centers of the DC and the SC immediately above the center of mass of the DC is established with a fixed probability (0.999), a mechanism similar to what has been proposed for keratinocytes (Czirók et al., 2013). Such links are cut whenever a DC loses contact with the WL, or the link reaches twice its original length, or with a small constant probability ($3x10^{-4}$). These rules allow the relative sliding of the deep and superficial layers.

Each SC and DC maintain an individual internal cell-cycle clock that is initiated at random and makes sure that each such cell undergoes exactly one cell division during the course of the simulations. The number of divisions is based on a previous study (Keller, 1980) and our current observation of in vivo time-lapse recordings that show that SCs divide approximately once or twice in the two-dimensional plane during epiboly. Assuming that there is no privileged orientation of divisions within the plane of the tissues (Longo et al., 2004, Woolner and Papalopulu, 2012), cells should divide once in the plane of our quasi-2D setup and once perpendicular to it. The latter would be unnoticed in our simulations; therefore cells in the model divide only once. Mitotic spindle orientation in SCs is always parallel to the plane of the SL following experimental observations (Longo et al., 2004, Woolner and Papalopulu, 2012). In our model DCs divide along their minor cell axis. Before cell division all connections are removed and automatically regenerated

following the same rules as above. In case of epithelial links (SC-SC links), the daughter cells are connected to each other and to one of the former connection partner of the parent nearest to them.

To achieve neutral boundary conditions that allow free expansion of the tissue but still prevent it from collapsing into a compact cluster, anchor points were introduced near the simulation sides above the WL. Anchor points, similar to WCs, are fixed (frozen) cells with a minimal size. SCs touching an anchor point form an "epithelial" connection with it, as described above. SCs that have their center of mass outside the anchor points and are not connected to an anchor point are considered outside the simulation area. Additionally, if all epithelial neighbors of a SC are connected to an anchor point, it is also considered to be outside. SCs outside the simulation are disconnected from all other cells and lose their volume constraint (but cannot expand over their original size). Similarly, deep cells that are outside the anchor points and are not connected to any SCs, lose their volume constraint, their ability to connect to any cell, and their ability to chemotax. Since outside cells lack volume constraint, they are free to disappear under pressure, but cannot re-enter the simulation (Voss-Böhme, 2012). In case no DCs are present under the anchor point in a 5x5 lattice site area, a new DC is introduced at this position. Anchor points are set at 30 lattice sites distance from the sides of the total simulation arena width of 400 lattice sites.

As expansion occurs by displacement of the whole tissue, mechanical equilibrium is required to prevent artificial wrinkles produced by a low speed of sound in the model (Szabó et al., 2012). This effect is overcome by introducing sufficiently high time resolution (Szabó et al., 2012). Therefore, we simulate 1 minute of real time using 600 computational time steps (Monte Carlo steps, MCS). Spatial calibration is based on relating the average cell size of 40µm to the simulated cell size of 8 lattice sites resulting in 5µm / pixel resolution. Using this calibration, the diffusion coefficient of the chemoattractant is set to 0.68, relating to $1.7x10^{-10}$ $m^2/s$ measured for Sdf-1 in cell culture medium (Lin and Butcher, 2006), and the decay rate is set to $4x10^{-5}$, relating approximately to a typical 1h protein half-life (Belle et al., 2006). For simulations of the in vitro chemotaxis assay a time calibration of 1 min = 10MCS was used, as in this experimental assay the cells are attached to the underlying substrate and the culture is equilibrated quicker. For these simulations parameters were scaled accordingly to keep the same real values, therefore diffusion parameter is set to 40 and decay rate is set to $2.4x10^{-3}$, and a simulation area of 400x400 lattice sites was used with two clusters of 23 cells initialized with their centers 100 lattice sites apart, giving rise to an approximate 50 lattice site gap between the clusters. Adhesion parameters in all simulations were set to provide adhesive SCs: $J_{SC,SC} = 2$, $J_{SC,medium} = 3$; slightly adhesive DCs: $J_{DC,DC} = 1.8$, $J_{DC,medium} = 1$; adhesive WCs: $J_{WC,DC} = J_{WC,SC} = 1$. Initial cell volumes are set to 64 lattice sites, chemotaxis saturation coefficient is set to $s=0.1$ (Eq. 4). The relative importance of the probability terms are set to: $\lambda_V=1$, $\lambda_c=100$, $\lambda_{i,j}=1$ for SC-DC connections and $\lambda_{i,j}=50$ for SC-SC connections.

## Supplemental References


Belle, A., Tanay, A., Bitincka, L., Shamir, R., and O'Shea, E.K. (2006). Quantification of protein half-lives in the budding yeast proteome. Proc. Natl. Acad. Sci. 103, 13004–13009.

Glazier, J.A., and Graner, F. (1993). Simulation of the differential adhesion driven rearrangement of biological cells. Phys. Rev. E 47, 2128–2154.

Harland, R.M. (1991). Xenopus laevis: Practical Uses in Cell and Molecular Biology (Elsevier).

Hossain, M.J., Whelan, P.F., Czirók, A., and Ghita, O. (2011). An active particle-based tracking framework for 2D and 3D time-lapse microscopy images. Conf. Proc. ... Annu. Int. Conf. IEEE Eng. Med. Biol. Soc. IEEE Eng. Med. Biol. Soc. Annu. Conf. 2011, 6613–6618.

Lin, F., and Butcher, E.C. (2006). T-cell chemotaxis in a simple microfluidic device. Lab Chip 6, 1462.

Nandadasa, S., Tao, Q., Menon, N.R., Heasman, J., and Wylie, C. (2009). N- and E-cadherins in Xenopus are specifically required in the neural and non-neural ectoderm, respectively, for F-actin assembly and morphogenetic movements. Development 136, 1327–1338.

Ramos, J.W., and DeSimone, D.W. (1996). Xenopus embryonic cell adhesion to fibronectin: position-specific activation of RGD/synergy site-dependent migratory behavior at gastrulation. J. Cell Biol. 134, 227–240.

Szabó, A., and Merks, R.M.H. (2013). Cellular potts modeling of tumor growth, tumor invasion, and tumor evolution. Front. Oncol. 3, 87.

Thirusittampalam, K., Hossain, M.J., Ghita, O., and Whelan, P.F. (2013). A Novel Framework for Cellular Tracking and Mitosis Detection in Dense Phase Contrast Microscopy Images. IEEE J. Biomed. Heal. Informatics 17, 642–653.

Voss-Böhme, A. (2012). Multi-Scale Modeling in Morphogenesis: A Critical Analysis of the Cellular Potts Model. PLoS One 7, e42852.
