# Supplementary figures and images for: The Molecular Basis of Radial Intercalation during Tissue Spreading in Early Development
Source: Dev Cell. 2016 May 9;37(3):213–25. doi: 10.1016/j.devcel.2016.04.008 (PMC4865533; doi:10.1016/j.devcel.2016.04.008)

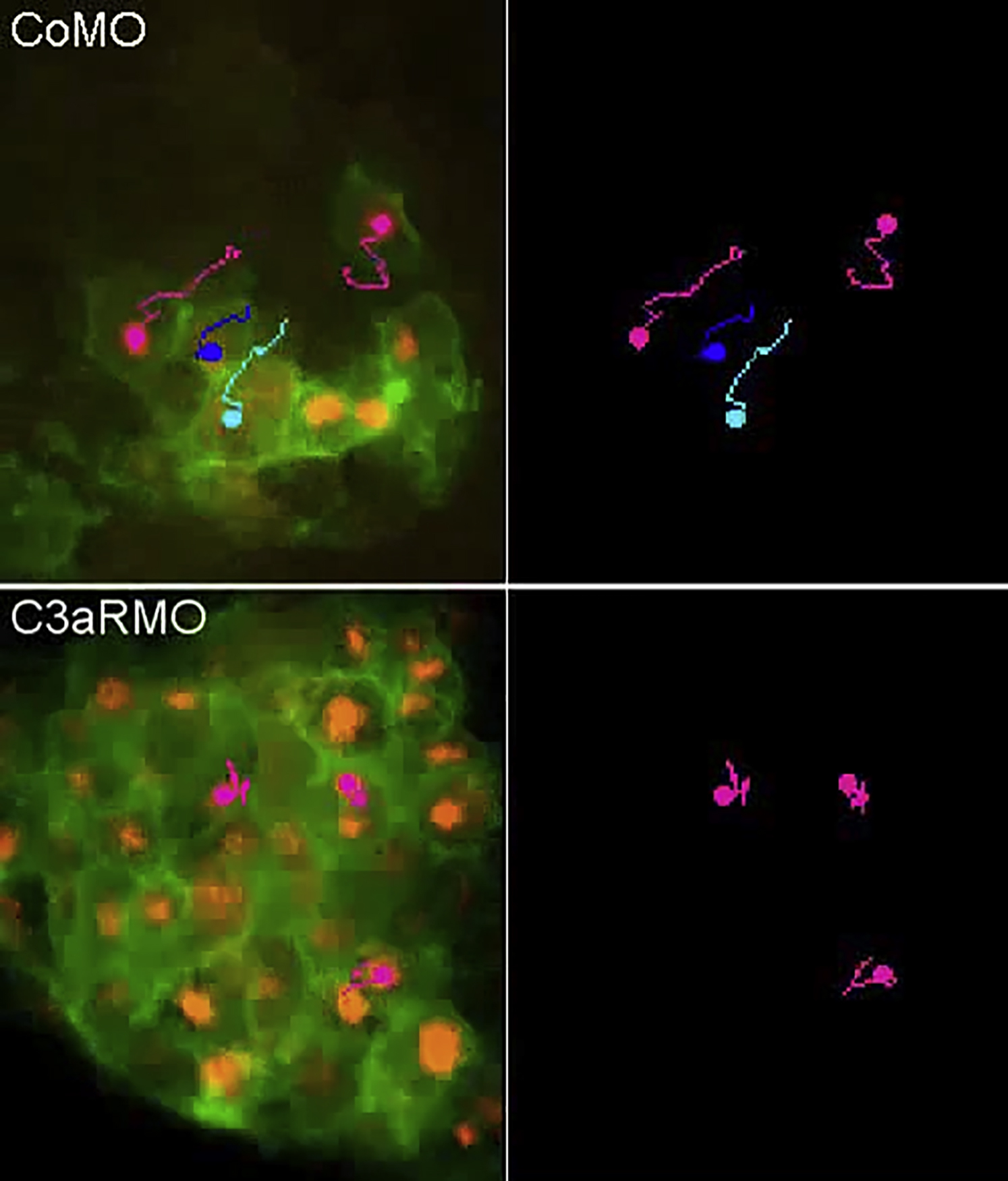

Supplement: Movie S1. Ex Vivo Assay of Radial Intercalation [file mmc2.jpg]

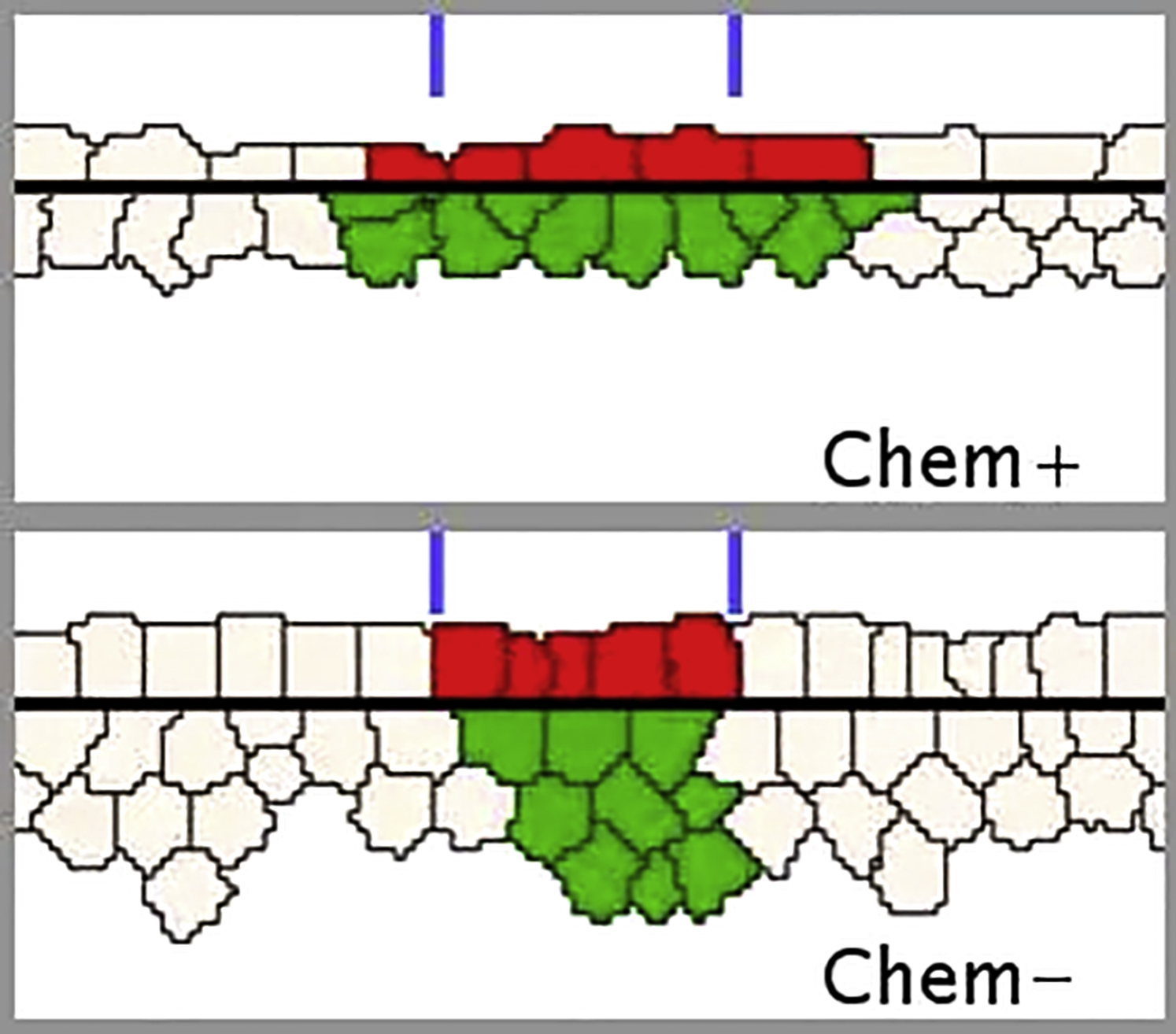

Supplement: Movie S2. Computational Model Simulation of Radial Intercalation [file mmc3.jpg]

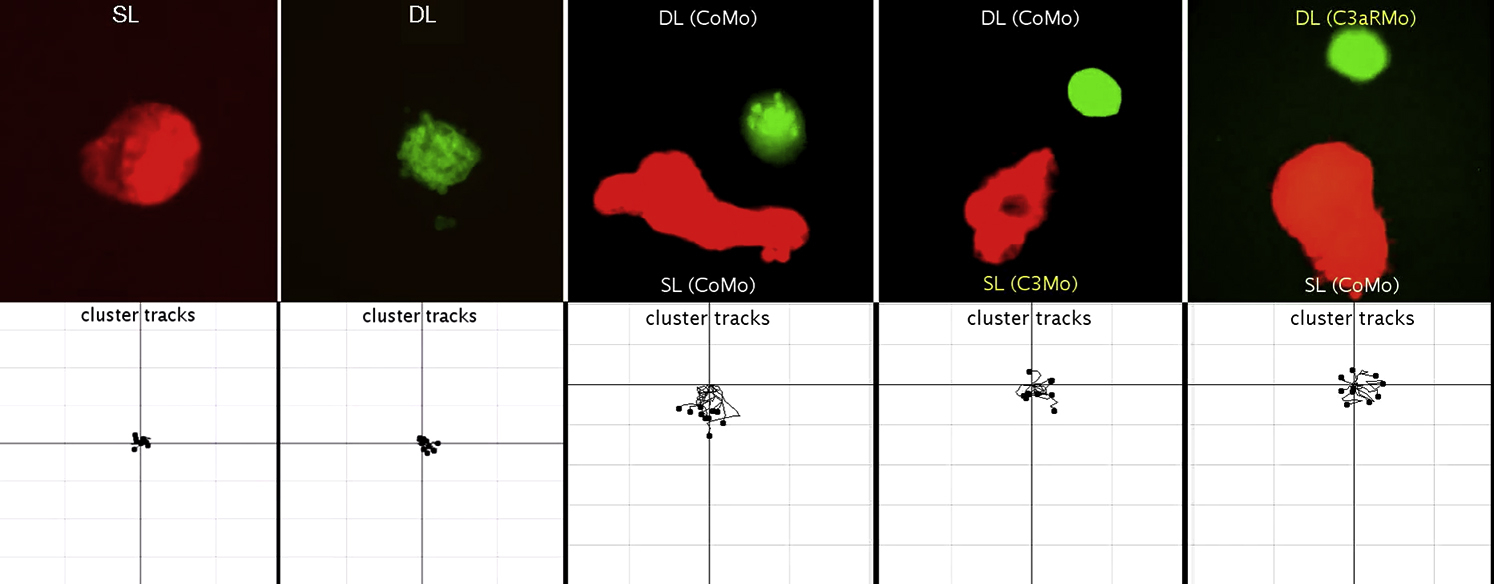

Supplement: Movie S3. In Vitro Chemotaxis Assay Using Deep and Superficial Layer Explants [file mmc4.jpg]

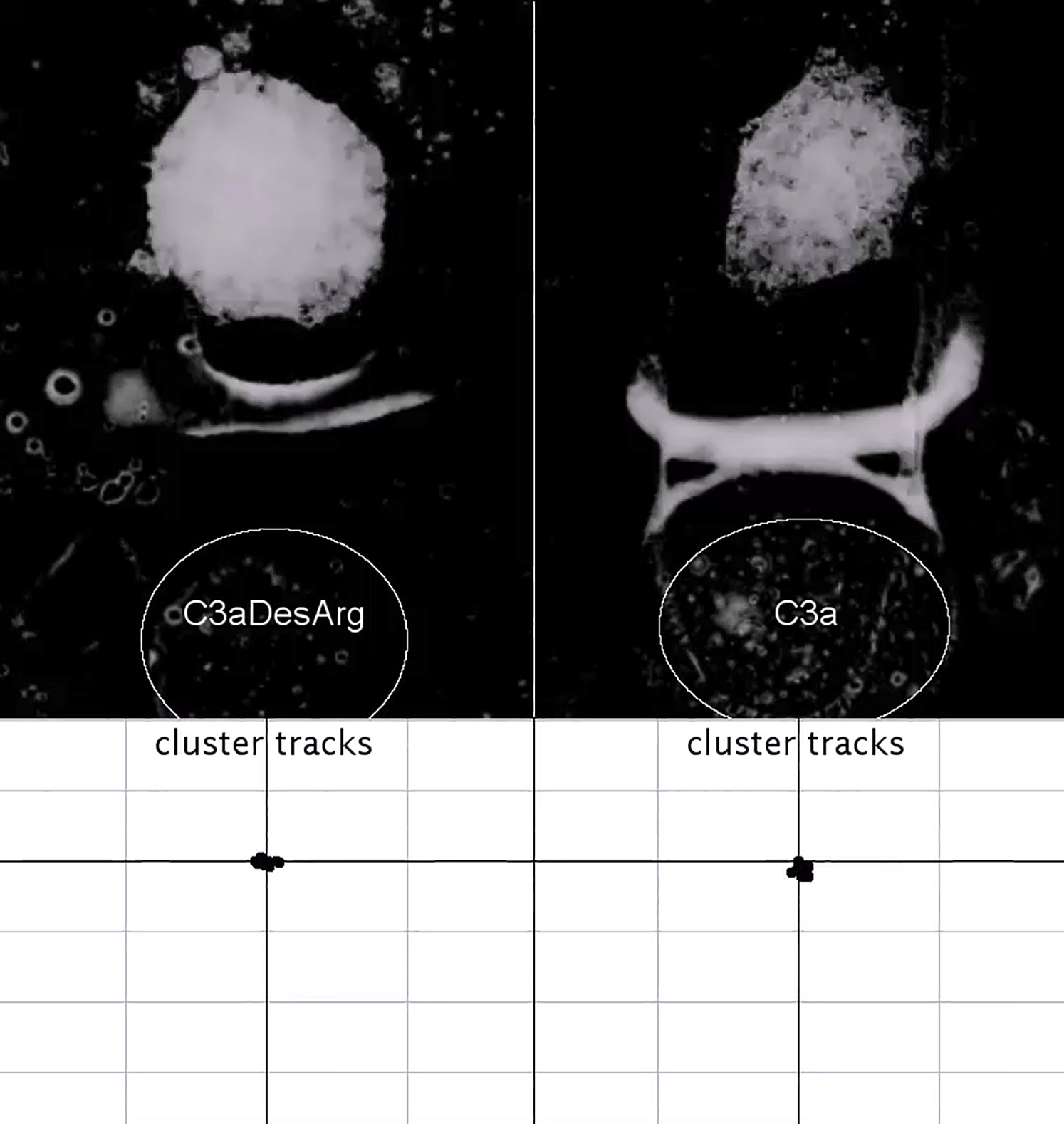

Supplement: Movie S4. In Vitro Chemotaxis Assay Using Deep Layer Explants and Purified C3a [file mmc5.jpg]

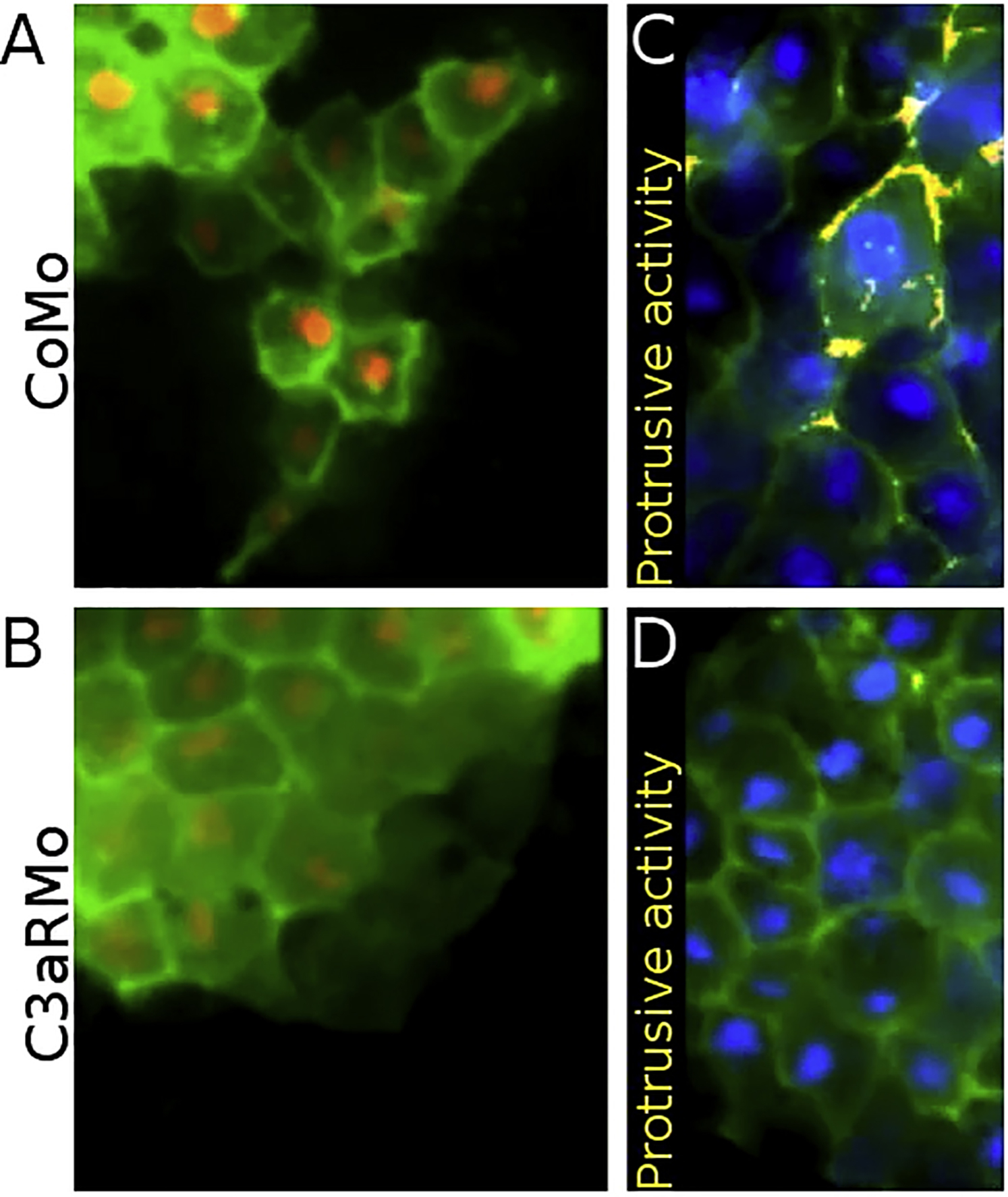

Supplement: Movie S5. Ex Vivo Assay Showing Protrusive Activity [file mmc6.jpg]

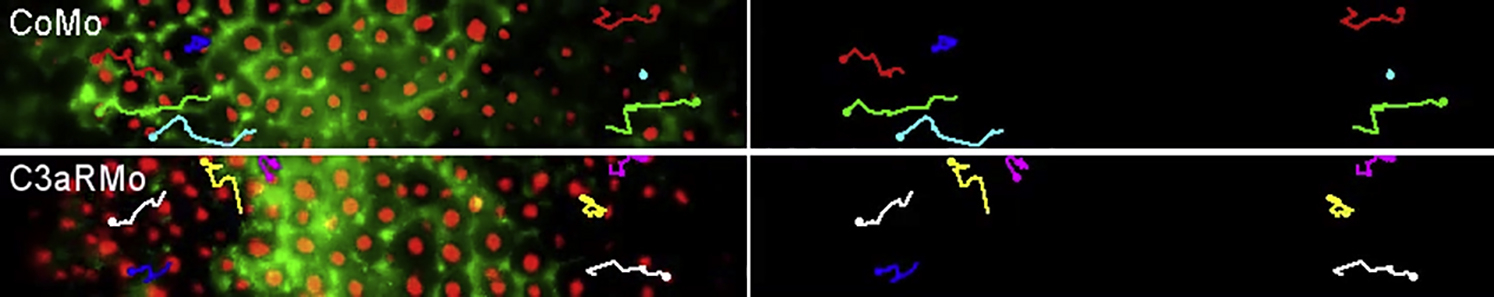

Supplement: Movie S6. In Vivo Expansion of the Blastocoel Roof during Epiboly [file mmc7.jpg]

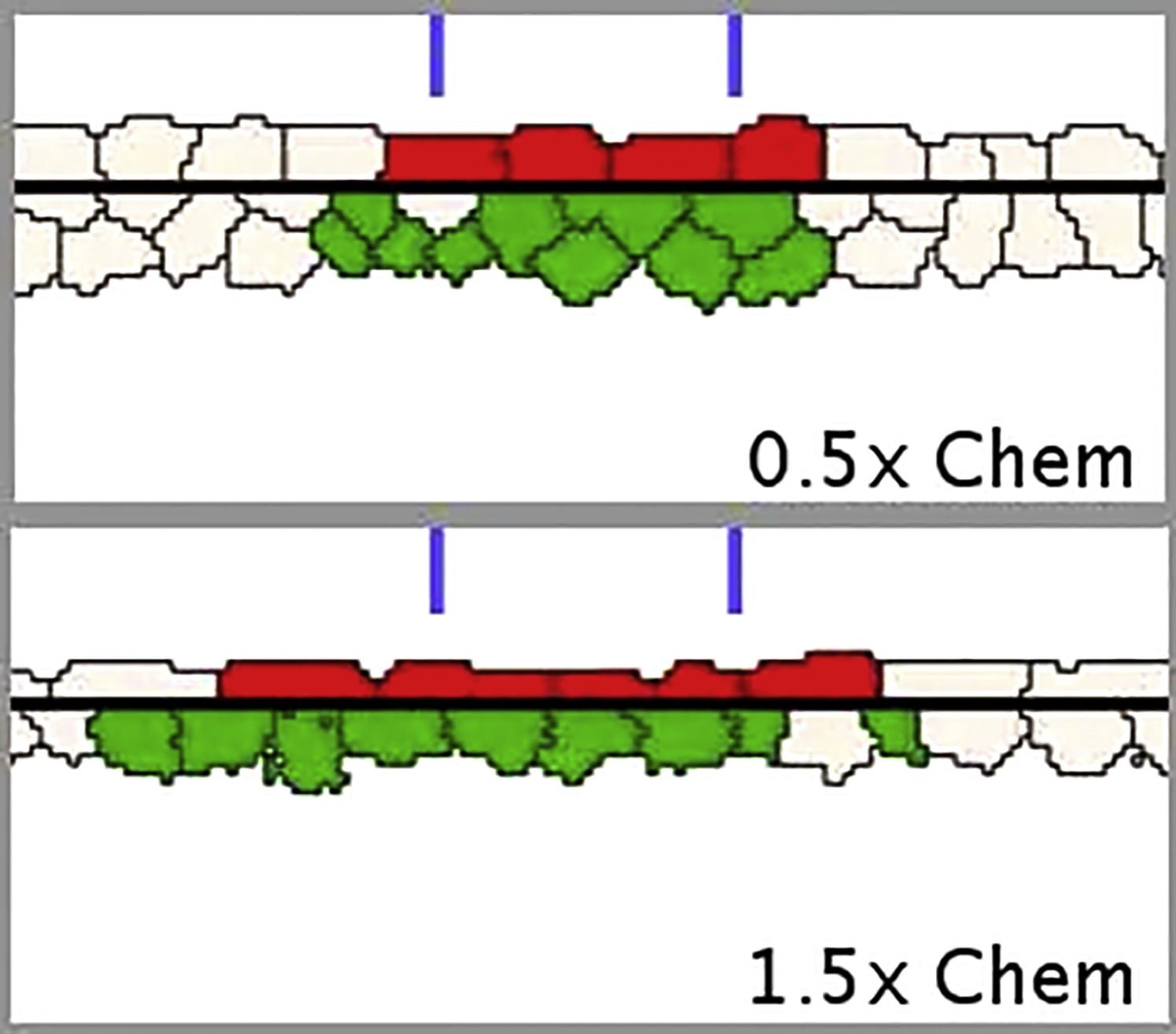

Supplement: Movie S7. Computational Model Simulation with Altered Chemoattractant Levels [file mmc8.jpg]
