# Supplementary material for: The Molecular Basis of Radial Intercalation during Tissue Spreading in Early Development
Source: Dev Cell. 2016 May 9;37(3):213–25. doi: 10.1016/j.devcel.2016.04.008 (PMC4865533; doi:10.1016/j.devcel.2016.04.008)
Supplement: Document S2. Article plus Supplemental Information [file mmc9.pdf]

Developmental Cell

Article

The Molecular Basis of Radial Intercalation during Tissue Spreading in Early Development

Graphical Abstract

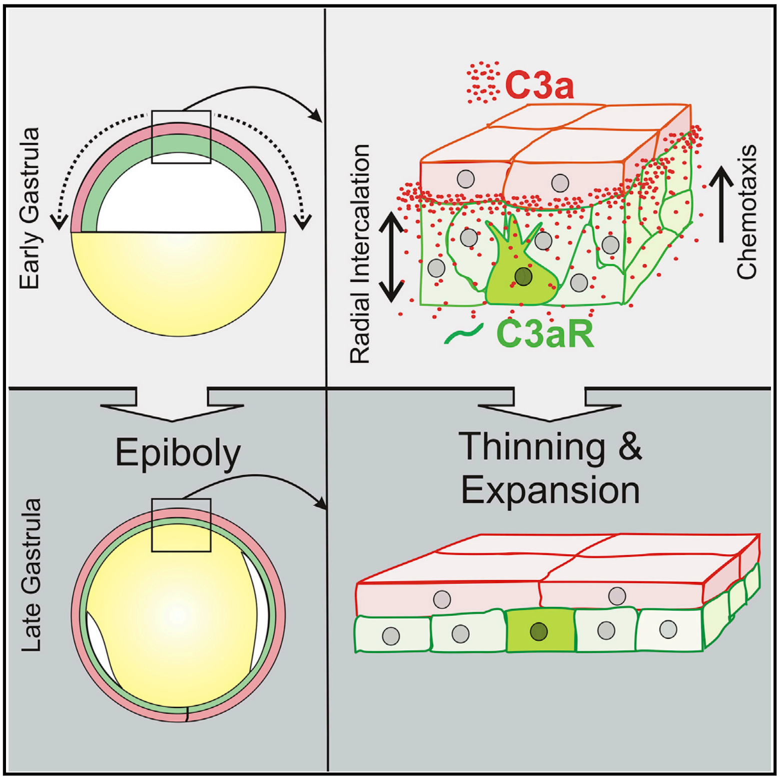

Highlights

- Radial intercalation is driven by short-range chemotaxis during epiboly
- Deep cells chemotax toward C3-producing superficial cells during *Xenopus* epiboly
- Radial intercalation is tissue autonomous and required for spreading
- Different levels of C3 expression explain dissimilarity in dorsoventral expansion

Authors

András Szabó, Isidoro Cobo, Sharif Omara, Sophie McLachlan, Ray Keller, Roberto Mayor

Correspondence

r.mayor@ucl.ac.uk

In Brief

Radial intercalation is responsible for the expansion and thinning of multilayered tissues during large-scale morphogenesis, such as epiboly during gastrulation. Focusing on amphibian epiboly, Szabó et al. show that intercalation is driven by short-range chemotaxis of cells toward the usually immune-associated complement component C3, produced by the external tissue layer.

Szabó et al., 2016, Developmental Cell *37*, 213–225
May 9, 2016 © 2016 The Authors. Published by Elsevier Inc.
http://dx.doi.org/10.1016/j.devcel.2016.04.008

CellPress

CellPress

The Molecular Basis of Radial Intercalation during Tissue Spreading in Early Development

András Szabó,[1,3] Isidoro Cobo,[1,3] Sharif Omara,[1] Sophie McLachlan,[1] Ray Keller,[2] and Roberto Mayor[1,*]
[1]Deparment of Cell and Developmental Biology, University College London, London WC1E 6BT, UK
[2]Department of Biology, University of Virginia, Charlottesville, VA 22908, USA
[3]Co-first author
*Correspondence: r.mayor@ucl.ac.uk
http://dx.doi.org/10.1016/j.devcel.2016.04.008

SUMMARY

Radial intercalation is a fundamental process responsible for the thinning of multilayered tissues during large-scale morphogenesis; however, its molecular mechanism has remained elusive. Using amphibian epiboly, the thinning and spreading of the animal hemisphere during gastrulation, here we provide evidence that radial intercalation is driven by chemotaxis of cells toward the external layer of the tissue. This role of chemotaxis in tissue spreading and thinning is unlike its typical role associated with large-distance directional movement of cells. We identify the chemoattractant as the complement component C3a, a factor normally linked with the immune system. The mechanism is explored by computational modeling and tested in vivo, ex vivo, and in vitro. This mechanism is robust against fluctuations of chemoattractant levels and expression patterns and explains expansion during epiboly. This study provides insight into the fundamental process of radial intercalation and could be applied to a wide range of morphogenetic events.

INTRODUCTION

Acquiring shape and form in multicellular organisms involves deformation of epithelial sheets through bending (invagination), extension through narrowing (convergent extension), and expansion via thinning (epiboly). During epiboly, the number of cell layers in a multilayered epithelium is reduced by cell intercalation, a process called radial intercalation (RI). RI was first described during the uniform expansion of the ectoderm in the animal pole region during amphibian gastrulation (Keller, 1980). Since then it has been recognized as a general morphogenetic process involved in a wide range of systems, including fish epiboly (Warga and Kimmel, 1990), fly gastrulation (Clark et al., 2011), amphibian and fish neural folding (Kee et al., 2008), regeneration of hydra (Kishimoto et al., 1996), and in mammalians during gastrulation (Yen et al., 2009), gut development (Yamada et al., 2010), and ear development (Chen et al., 2002). Two main mechanisms have been proposed so far to explain RI. Studies of amphibian epiboly proposed that RI is driven by adhesion to a fibronectin matrix accumulated on the basal surface of the ectoderm allowing protrusive activity only at the fibronectin-free cell surfaces (Marsden and DeSimone, 2001; Petridou et al., 2013; Sugrue and Hay, 1981). However, this would not explain the intercalation of cells that are not in direct contact with the fibronectin. Studies of teleost epiboly propose cell sorting via differential cell adhesion as the driving force behind RI (Kane et al., 2005; Málaga-Trillo et al., 2009; Schepis et al., 2012). A gradient of cell-cell adhesion molecules toward the superficial surface would drive ectodermal cells to move in the direction of the gradient, opposite to what the fibronectin hypothesis would predict (Kane et al., 2005; Málaga-Trillo et al., 2009; Schepis et al., 2012). Although both cell-matrix and cell-cell adhesions are likely to be involved in some capacity, it is beyond doubt that other mechanisms are required to fully explain this process.

Using *Xenopus laevis*, the original model system to study RI, here we propose a mechanism for epiboly in which the rearrangement of ectodermal cells is driven by an unexpected activity of complement component C3, a factor normally associated with the immune system (Ricklin et al., 2010; Leslie and Mayor, 2013). In immune cell homing, C3 is cleaved to produce C3a, a small anaphylatoxin peptide that binds to C3aR and triggers chemotaxis (Leslie and Mayor, 2013). We developed a computational model to test the hypothesis that RI is driven by deep cell (DC) chemotaxis toward superficial ectoderm cells (SCs). Several predictions of the model were tested in vivo, ex vivo, and in vitro, confirming the notion that short-range chemotaxis is required for ectoderm epiboly.

RESULTS

Radial Intercalation during *Xenopus* Epiboly Is Accompanied by C3 and C3aR Expression

Epiboly in *Xenopus* embryos takes place between developmental stages 8 and 11.5 during which the blastocoel roof (BCR) of the animal pole region expands and thins. At the onset of epiboly, the BCR consists of a tightly connected superficial epithelial monolayer (SL; Figures 1A and 1B, red), and a multilayered deep layer (DL; Figures 1A and 1B, green) comprising 2–4 tiers of loosely connected DCs (Keller, 1978, 1980). During epiboly, DCs undergo RI to form a single cell layer (Figure 1B; Keller, 1980), but they do not invade the SL (Keller, 1978). SCs

This is an open access article under the CC BY license (http://creativecommons.org/licenses/by/4.0/).

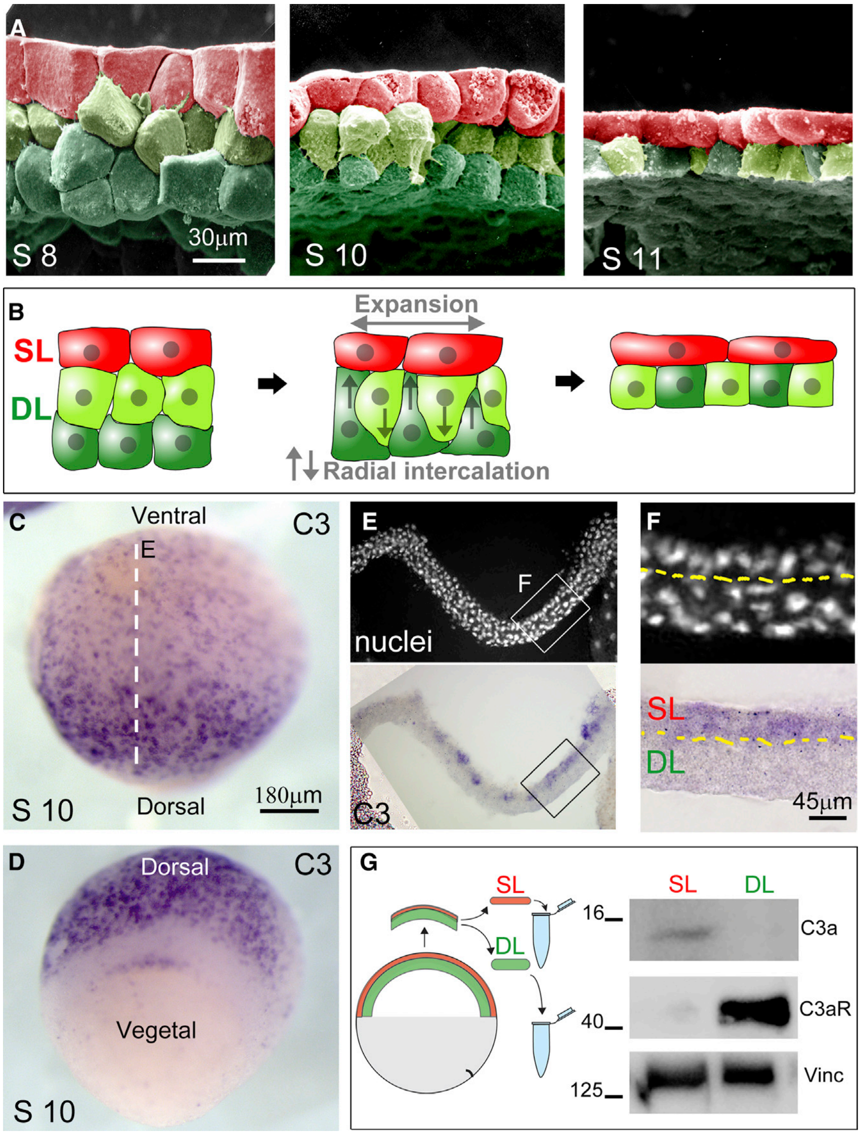

Figure 1. Radial Intercalation Is Accompanied by Expression of C3 and C3aR during *Xenopus* Epiboly

(A) SEM images of blastocoel roof during epiboly in *Xenopus* embryos at stages 8, 10, and 11. False coloring indicates superficial cells (red), and the intercalation of outer-deep (light green) and inner-deep (dark-green) cells.

(B) Schematic illustration of the process of epiboly in *Xenopus* shown in (A), including expansion of the superficial layer (SL) and the RI of the deep cell layer (DL). Note that the DL does not intercalate into the SL.

(C and D) In situ hybridization (ISH) reveals C3 expression in the animal (C) but not in the vegetal (D) pole region during epiboly.

(E and F) Cross-section (E) and zoom (F) of nuclei and ISH along the dashed line indicated in (C) show that C3 is expressed in the SL.

(G) Western blot analysis showing differential expression of C3a and C3aR in the SL and DL of stage 10 embryos, respectively. Loading control, vinculin.

change from cuboidal to squamous shape as the whole tissue expands.

In embryos undergoing epiboly, the complement component C3 is expressed at the expanding animal pole region (Figure 1C; in situ hybridization [ISH]) while absent in the ingressing vegetal pole (Figure 1D). Cross-sections along the BCR region indicate that C3 is specifically expressed in the SL (Figures 1E and 1F). Western blot analysis of separated SL and DL samples from stage 10 embryos shows the specificity of the C3a peptide to the SL and the presence of its cognate receptor C3aR in the DL (Figure 1G).

C3 and C3aR Are Required for Radial Intercalation

To investigate the role of C3 and C3aR on RI, we analyzed the number of cell layers of the BCR in loss-of-function experiments using antisense morpholino oligonucleotides (Mos) against C3aR (C3aRMo; Carmona-Fontaine et al., 2011), C3 (C3Mo; see Supplemental Information for controls) or antibodies against C3a (C3aAb; Carmona-Fontaine et al., 2011). Treatments left the tissue geometry unaffected prior to RI (Figure 2A) but led to a higher number of cell layers after epiboly when compared with control (Figures 2B and 2C) and eventually led to failure of blastopore closure and altered internal embryo structure (Figure S1). Importantly, control and rescue experiments and analysis of protein levels establish the specificity of these blocking reagents (Figure S2). Further characterization of these inhibitors shows that all of them produce the same phenotype, and therefore here we describe only one of them (C3aR depletion), except where mentioned otherwise.

It has been suggested that cell-cell adhesion (Kane et al., 2005; Málaga-Trillo et al., 2009; Schepis et al., 2012) and adhesion to fibronectin (Marsden and DeSimone, 2001; Petridou et al., 2013) are important for epiboly. To test if C3aR inhibition blocked epiboly by interfering with cell adhesion, we performed adhesion and sorting assays, finding no difference between control and C3aR-depleted cells (Figure S3). DC adhesion to fibronectin and fibronectin deposition in the BCR were found to be unaffected by the absence of C3aR (Figure S4). Germ layer specification, indicated by the expression of several known ectodermal and mesodermal markers, was normal after C3aRMo injection (Figure S5). In addition, C3aRMo injection had no observable effect on the cell size (17 ± 2 μm CoMo, 18 ± 6 μm C3aRMo) or number of cell divisions (1.3 ± 0.2 CoMo, 1.4 ± 0.2 C3aRMo, n = 200 cells). In conclusion, germ layer specification, cell adhesion, cell size, and proliferation are unaffected in embryos lacking C3aR.

To address whether the effect of C3aR inhibition on epiboly is independent of the SL, we blocked C3aR specifically in the DL by using a modified BCR explant culture (Marsden and

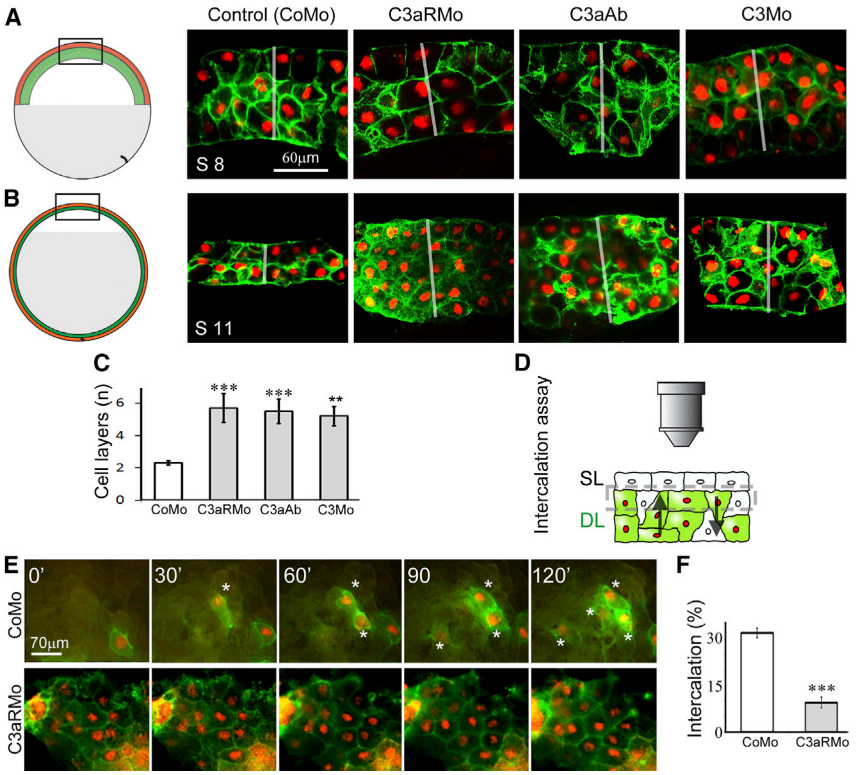

Figure 2. C3 and C3aR Are Required for Radial Intercalation

(A and B) Sections showing the blastocoel roof before (A, stage 8) and after (B, stage 11) epiboly in control and treated embryos. Embryos deficient in C3aR (C3aRMo), treated with C3a antibody (C3aAb), or suppressed C3 expression (C3Mo) remain thick and multilayered by the end of epiboly (red, nucleus; green, membrane; white bars indicate tissue thickness). In contrast, the tissue thins into a dual-layered epithelium in control (CoMo) embryos.

(C) The number of cell layers by the end of epiboly is increased with C3aRMo (n = 62), C3a antibody (C3aAb; n = 30), or C3Mo (n = 60) when compared with control embryos, indicating the lack of RI in the absence of C3 signaling.

(D) Schematic of the ex vivo intercalation assay showing the explant from the side. A dashed rectangle indicates the focal range of imaging, which includes the top region of the DL that is in close vicinity of the SL.

(E) Frames from time-lapse recording in the ex vivo assay show intercalating DCs (*) in a control (CoMo) and C3aR-deficient (C3aRMo) tissue explants.

(F) The number of DCs that intercalate is significantly lower in C3aRMo tissues than in control tissues, showing that intercalation is hampered in the absence of C3aR signaling.

Data are represented as means ± SD, t test significances are ***$p < 0.001$, **$p < 0.01$. See also Figures S1–S5.

DeSimone, 2001). Briefly, the DL from a mosaic labeled embryo was cultured on a dish underneath an SL from an unlabeled control embryo and imaged from above using time-lapse microscopy. The unlabeled SL allows imaging of the apical (top) portion of the DL in close vicinity of the SL (Figure 2D). In this ex vivo assay, intercalation of DCs is observed as newly emerging or disappearing cells, as these cells enter or leave the top portion of the DL (Figure 2E, asterisks mark intercalation; Movie S1, blue tracks). The ratio of such intercalating cells and cells that do not leave the visualized plane (intercalation percentage) in C3aRMo explants is severely reduced compared with the ratio found in CoMo explants, which indicates that C3aR activity is directly involved in RI at the BCR independently of the SL (Figure 2F).

Radial Intercalation Driven by C3 Chemotaxis

A well-characterized activity of C3a is to promote chemotaxis during immune response through binding to its receptor C3aR (Leslie and Mayor, 2013). C3a/C3aR induce a similar function during development, whereby they promote cohesion through autocrine chemotaxis of the migrating neural crest cells (Carmona-Fontaine et al., 2011). Based on these observations, we hypothesized that RI is driven by close-range chemotaxis of the DCs toward the C3a producing SCs (Figure 3A).

We turned to computational modeling to test this hypothesis. We implemented a cellular Potts model (Graner and Glazier, 1992) with a quasi-2D cross-section of the BCR including an SL in which all SCs secrete a chemoattractant (Figures 3A and 3B), resulting in an emergent gradient (Figure S6A). ISH shows a mosaic expression of C3 (Figure 1C), therefore we also tested an alternating production pattern in the SL and found that the emerging chemoattractant gradient is largely insensitive to the secretion pattern (Figures S6B–S6D). As both patterns of expression generate the same result in the model, we only show a uniform secretion pattern for the rest of the study. DC chemotaxis is implemented by increasing the probability of cell displacements toward higher concentrations of the chemoattractant (Merks et al., 2004). Upon reaching the SL, DCs form an elastic bond with their SC neighbors with a probabilistic rule that allows for dynamic cell-cell adhesions. Such bonds have been shown to control tissue viscosity (Czirók et al., 2013) and may describe any mechanical resistance of the cells to change their distance, including but not limited to cell adhesion. During the course of the simulated 4 hr real time, all cells undergo one division without cell growth which corresponds to two divisions in the 2D tissue (Keller, 1980). For more details, see Supplemental Information.

Starting from two layers of DCs (Figure 3B, green; shades are only visual aid) and a single SC layer (red), the simulated tissue thins to produce a single layer of DCs via RI. In the absence of chemotaxis, intercalation is severely reduced and the average thickness of the tissue remains higher than with chemotaxis (Figures 3B and 3C; Movie S2). This change in thickness is comparable with the change observed in the BCR thickness in vivo at the end of epiboly in control (CoMo) or C3aR/C3-inhibited (C3aRMo, C3aAb, C3Mo) embryos (Figure 3D). Total tissue thickness in silico is largely insensitive to the cohesion between the DL and SL, which only influences the thickness ratio of the SL and DL (Figure S6E).

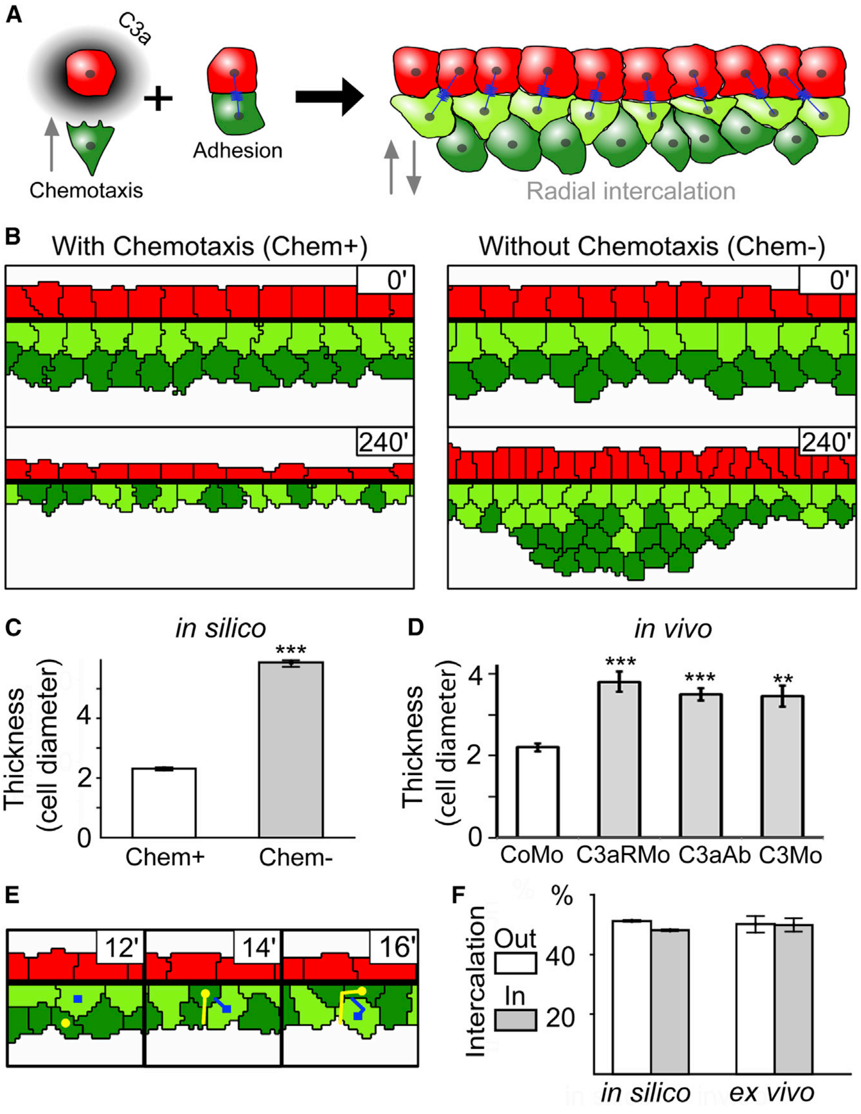

Figure 3. Chemotaxis-Based Radial Intercalation Hypothesis and Computer Simulation

(A) Hypothesis: chemotaxis of DCs (green) toward SCs (red) via C3a drives RI and consequent tissue thinning.

(B) Computer simulations of the hypothesis showing the initial (0′) and final (240′) cell configurations with chemotaxis (Chem+) and without chemotaxis (Chem-). Coloring as in Figures 1A and 1B, for visual aid only.

(C) Tissue thickness, measured in units of cell diameter after epiboly (20 μm), is significantly increased in simulations without chemotaxis (n = 30).

(D) In vivo tissue thickness, measured in units of cell diameter after epiboly (20 μm), is significantly higher in embryos with either impaired C3 receptors (C3aRMo, n = 62) or C3 ligands (C3aAb, n = 35; C3Mo, n = 62) compared with control embryos (CoMo).

(E) Prediction of bidirectional intercalation. In silico, DCs intercalate both toward the secreting SL, driven by chemotaxis, and against the chemotactic gradient away from the SL, driven by volume exclusion.

(F) In silico DC movement toward the SL (Out) is only slightly more frequent than movement away from it (In). Direction of DC intercalation measured in the intercalation assay (ex vivo, see Figure 2) confirms the model's prediction.

Error bars: SD, significance **$p < 0.01$, ***$p < 0.001$. See also Figure S6.

Although chemotaxis actively drives DCs toward the SL, the model predicts passive cell movement in the opposite direction as well (Figure 3E). The percentage of DC movement toward the SL (Out, 51.3% ± 0.2%) is only slightly higher in silico than movement away from the SL (In, 48.1% ± 0.1%; Figure 3F). We tested this prediction using the ex vivo assay (Figure 2D), where the ratio of DCs intercalating toward the SL (appearing, light blue tracks in Movie S1) and DCs intercalating away from the SL (disappearing, dark blue track in Movie S1) showed a similar bidirectionality (Out, 50% ± 3%; In, 50% ± 2%; Figure 3F). Movement away from the SL is explained by volume exclusion in silico, but we cannot rule out other mechanisms ex vivo, such as repolarization upon contact (Höpker et al., 1999). These results, together with recent in vivo data on RI during zebrafish epiboly showing bidirectional intercalation (Bensch et al., 2013), confirm the bidirectional movement predicted by the computational model.

Taken together, our in silico results support the hypothesis that RI is driven by chemotaxis. Next, we explore the experimental foundation for the chemotactic interaction.

Testing C3 Chemotaxis of the Deep Cells

To test chemotaxis, the basic assumption of our hypothesis, we utilized a classical chemotaxis assay by culturing deep and superficial explants at a distance (Figure 4A). Our computational model predicts that, in the presence of chemotaxis, the DL explant will move toward the SL explant and, in the absence of chemotaxis, the clusters remain stationary (Figures S6F–S6G). Next, we cultured and tracked different combinations of DC and SC explants in vitro. In line with the predictions, the otherwise non-motile DC explants exhibit directional movement toward non-motile SCs cultured nearby (Figures 4B–4D; Movie S3; see also Figures S6H and S6I for persistence and speed). This movement is compromised when C3 is inhibited in SCs or when C3aR is inhibited in DCs (Figures 4E and 4F), showing that chemotaxis of the DCs toward the SCs is dependent on the C3a/C3aR axis, and that the amount of C3a produced by the SCs is sufficient for chemotaxis. The specificity of C3a in this chemotaxis is shown by the directional movement of DC explants toward a localized source of purified C3a, but not toward a mutant form of C3a (C3aDesArg) that is unable to bind the receptor (Honczarenko et al., 2005) (Figures 4G–4I; Movie S4). As summarized by the chemotaxis indices (Figure 4J), we conclude that DC chemotaxis toward SCs is C3a/C3aR dependent, in line with the in silico measurements (Figure 4K). In vivo, the short distance between the SL and DL could allow a similar chemotaxis to occur in spite

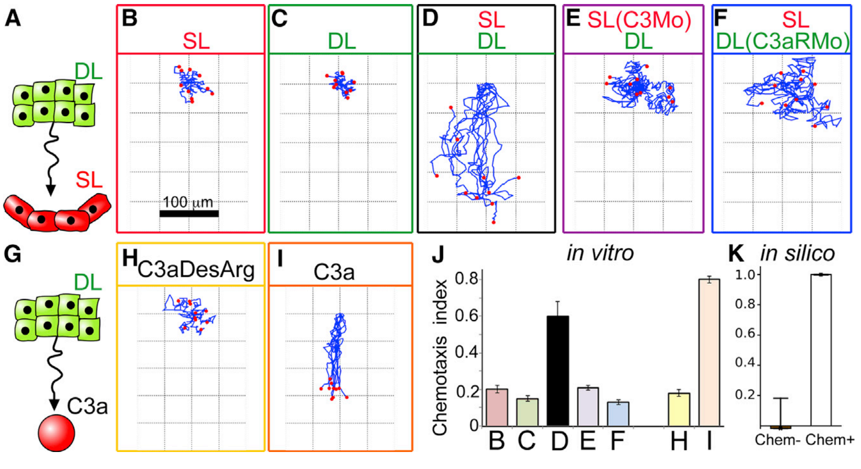

Figure 4. C3a-Based Chemotaxis between Cells of the Deep and Superficial Layers

(A–K) Testing the basic assumption of the model. Classical chemotaxis assay using DL and SL explants (A), or DL explants with purified C3a protein (G). Trajectories of SL (B) and DL (C–F, H, and I) explants, and DL chemotaxis toward non-functional (C3aDesArg, H, n = 35) and functional (I, n = 38) C3a source with corresponding chemotaxis indices in vitro (J) and in silico (K). Error bars: SD. See also Figure S6.

of the apparently low levels of C3a detected in the SCs by western blot (Figure 1G).

Such behavior in vivo is expected to generate a higher number of cell protrusions on the sides of DCs facing the SL. Direction of DC protrusions measured on scanning electron microscopy (SEM) images of fixed and fractured embryos during gastrulation revealed protrusions in all directions but with a very strong bias toward the SL (Figures 5A–5E and S6J). This finding suggests that chemotaxis is also present in vivo during the RI of the BCR.

Dynamic protrusive activity at the DL surface proximal to the SL was observed in our ex vivo assay, where the top portion of the DL explant is imaged from above through the SL (Figures 5F–5N). Protrusion formation was apparent in CoMo explants where stained DCs are adjacent to unlabeled DCs (Figure 5F, arrowheads). Protrusions were less frequent and smaller in C3aRMo explants (Figure 5G; Movie S5). Protrusions were quantified using the protrusion-retraction method (Scarpa et al., 2015). The area of these protrusions shows a significantly higher protrusive activity in control (CoMo) versus treated (C3aRMo) explants (Figures 5I and 5J). DL explants were imaged solely in the proximity of the SL, therefore any protrusion is indicative of protrusive activity toward or nearby the SL (Figure 5K). Protrusive activity between labeled DCs of CoMo explants is detected as increased levels of membrane staining caused by the overlap of protrusions at the cell surface (Figure 5L, red pseudocoloring). The threshold for the membrane signal to be considered as protrusive activity is set to match the level of a tissue with quasi-static boundaries. Activity is reduced in C3aRMo-treated DL explants (Figure 5M; Movie S5), also shown by the reduction of protrusive activity area per cell (Figure 5N). In conclusion, protrusive activity analyzed by SEM or by live imaging shows that most of the protrusions of DCs point toward the SCs, which is consistent with chemotaxis of DL cells toward the SL.

In the following, we explore an implication of the model, and then study its behavior while perturbing the level and the localization of the chemoattractant.

Tissue Expansion Requires C3 Signaling

Our chemotaxis-based in silico model of RI predicts the expansion of the whole tissue. This expansion is concomitant with RI and depends on chemotaxis (Figures 6A–6C; Movie S2). DL expansion in our model is transmitted to the SL locally via an effective friction reducing the shear, relative movement between the SL and DL as a result of the elastic links between DCs and SCs. Consequently, the DL expands slightly more than the SL, which is in agreement with previous experimental observations (Keller, 1980; Bauer et al., 1994). We tested the prediction that expansion during epiboly depends on DC chemotaxis using time-lapse imaging of CoMo- and C3aRMo-treated embryos (Figure 6D). The expected expansion of the animal pole in CoMo embryos was observed through the increase in distance of SC pairs over time (Figures 6E; Movie S6). When RI was blocked using C3aRMo, SCs in the animal pole remained at an approximately constant distance from one another (Figure 6F), supporting the in silico prediction.

In order to test whether the interaction between the SL and DL is sufficient to drive this expansion independently of the rest of the embryo, we used our ex vivo assay where only the SL and DL are present (Figure 6G). We observed that the distance between DCs generally increased over time in CoMo explants but not in C3aRMo explants (Figures 6H and 6I), showing that the interaction between SL and DL is sufficient to drive expansion in a C3aR-dependent manner.

Expansion in all three systems (in silico, in vivo, and ex vivo) showed a significant reduction in the absence of chemotaxis or C3aR (Figure 6J). Expansion was quantified by measuring how the distance of tracked cell pairs changes during imaging. For a given cell pair, the expansion is $E = (d_f - d_i)/d_i$, where d_f is the final distance and d_i is the initial distance. Expansion of the tissue is the average of expansions for all measured cell pairs. We observed a slight expansion in the Mo-treated specimens, which is unexplained by our model. This expansion could result from any remaining functional C3aR due to incomplete blocking by the Mo in the experiments as opposed to perfect inhibition in the model. We cannot exclude the possibility of an alternative expansion mechanism acting independently of RI, such as cell-autonomous expansion in the SL. However, as the majority of expansion is lost upon C3aR inhibition, any alternative mechanism is expected to play a minor role during BCR epiboly.

Our simulations show that this mechanism could also drive thinning of tissues that have a higher number of cell layers than *Xenopus* ectoderm, such as zebrafish epiboly (Figure S7A). Furthermore, we simulated experiments in which DCs were depleted, showing that there was no major effect on tissue expansion (Figures S7B and S7C), indicating the robustness of our model. In conclusion, our combination of simulations and experiments show for the first time that RI is required for the

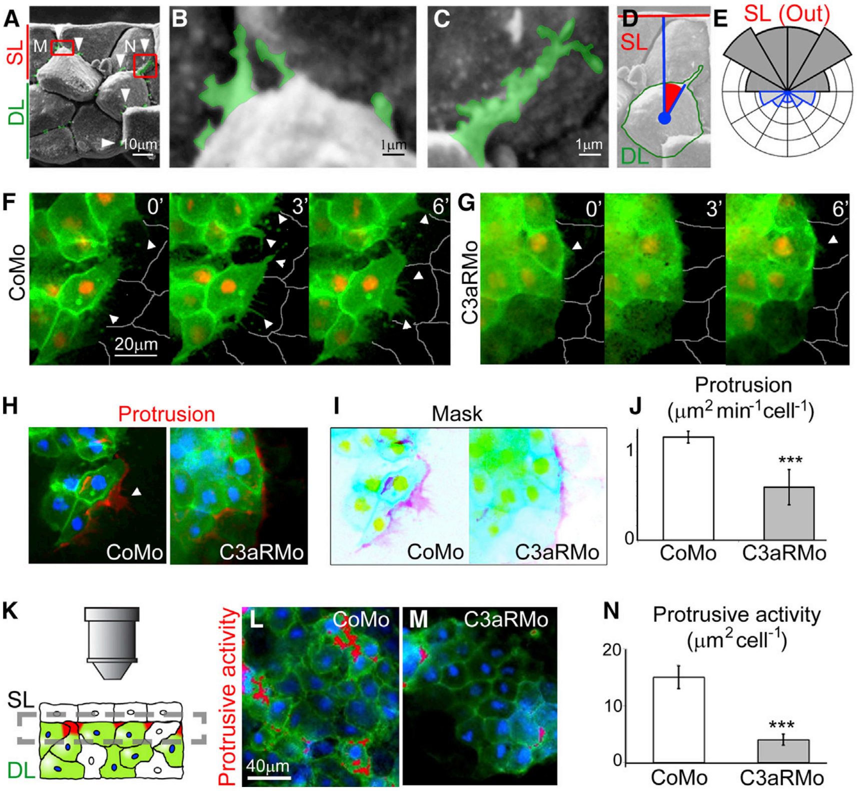

Figure 5. DC Protrusion Analysis

(A–E) Direction of DC protrusions in vivo (arrowheads, A–C) was measured in relation to the SL external surface (D) to reveal a bias toward the SL (E, n = 64 embryos, 1445 protrusions).

(F and G) Frames from time-lapse imaging showing DC protrusions (arrowheads) ex vivo (see Figure 2D). Protrusions are apparent in the vicinity of the SL in control (CoMo) but not in the C3aRMo-treated explants. Green, membrane; red, nuclei.

(H–J) Protrusion activity in the ex vivo assay analyzed using the extension-retraction method. Red shows the difference in membrane signal (green) between frames 3 min apart (H). The difference reveals the extending protrusions (I, purple).

(K–N) Ex vivo apical protrusive activity of DCs (L and M: green, membrane; red, protrusion; blue, nuclei) is decreased in C3aR-deficient explants (N: n = 32; ***$p < 0.01$; error, SD).

expansion of an adjacent tissue that does not participate in intercalation, and that a minimal of two DC layers are required for this process.

Chemoattractant Levels Modulate the Extent of Radial Intercalation and Extension

We investigated how the behavior of the model depends on the amount of chemoattractant in the system. Chemoattractant levels in the model are dynamically controlled by the secretion and decay rates, and the diffusion parameter, which together give rise to a quasi-steady concentration field. As these parameters are not directly accessible experimentally in vivo, here we measure the relative amount of chemoattractant produced in the SL in silico as a result of changing the secretion rate. A reduction of the produced chemoattractant levels to half did not impede RI, and tissue expansion was still visible in silico (Figure 7A, 0.5×). Increasing the chemoattractant levels by the same amount increased tissue expansion (Figure 7A, 1.5×; Movie S7). In silico tissue expansion increases rapidly even at low levels of chemoattractant but saturates at around triple concentration levels, where the tissue expands to slightly over double its linear size (Figure 7B, black). Tissue thickness similarly drops rapidly as the chemoattractant level is increased and reaches saturation (Figure 7B, red). Therefore the model predicts a slight reduction of epiboly at lower C3 expression levels but still noticeable epiboly even at levels as low as 10%.

To test this prediction, we analyzed C3 expression in subregions within the same embryo using ISH and found a considerable difference between the dorsal and ventral sides of the animal pole compared with the overall average in the whole-animal cap (Figures 7C and 7D). We found significantly higher expansion in the dorsal regions than in ventral regions as predicted by the model based on the same expression levels (Figures 7E and 7F). We conclude that this asymmetry in C3 expression might contribute to the known dorsoventral morphogenetic differences in gastrulation of *Xenopus* embryos (Bauer et al., 1994).

Localization of the Chemoattractant Source

Finally, we tested how the localization of the chemoattractant source within the tissue affects its morphogenesis. For this we compared simulations of normal chemoattractant expression (Control, Figures 8A and 8E) to simulations where no chemoattractant is present (Inhibition, Figures 8B and 8F), or is produced by all cells (Ubiquitous, Figures 8C and 8G), or is produced only in a restricted region of the SL (Localized, Figures 8D and 8H). As expected, inhibition resulted in thicker tissues than in controls. More surprisingly, a ubiquitous expression in the SL and DL resulted in tissue thinning similar to the control case in silico (Figure 8C). While a ubiquitous expression in uniformly packed tissues is expected to eliminate any chemotactic gradients, cell-free regions in our system take up a considerable volume. These regions represent the blastocoel cavity immediately below the DL and the external space above the SL. The chemoattractant diffuses from the cellular region into the cell-free area and is diluted in the comparatively large cavity of the blastocoel, giving rise to a gradient with decreasing concentration levels from the cellular region toward the blastocoel cavity (Figure 8G). Membranes of the DCs at the cavity edge fluctuate stochastically, extending into and retracting from the cavity. Membrane extensions into the cavity now occur against the generated chemotactic gradient, and therefore these are suppressed in the model. Through such a ratchet mechanism, this gradient directs cells from the edge of the blastocoel cavity toward the SL and promotes intercalation. In contrast, overexpression in

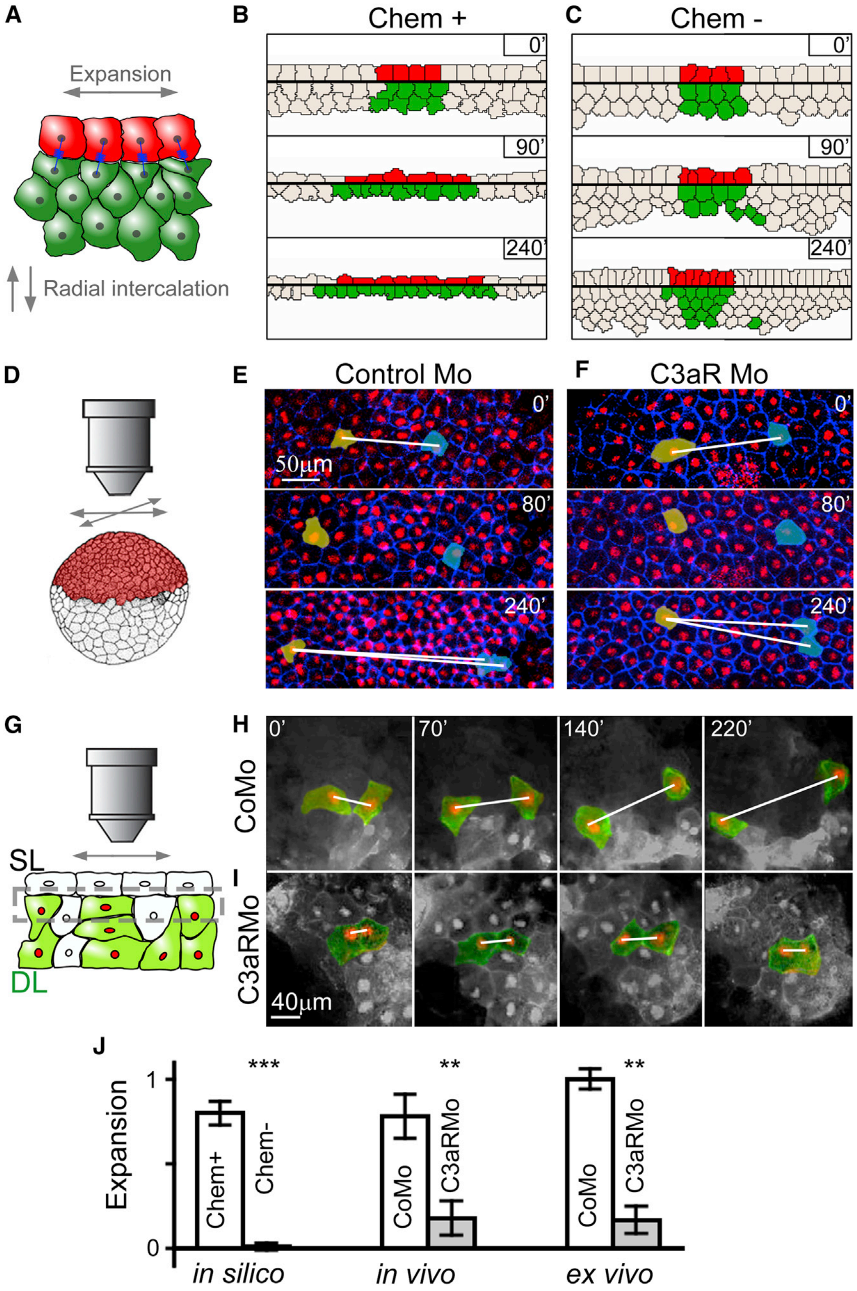

Figure 6. C3 Signaling Is Required for Epithelial Expansion

(A–C) Model prediction of tissue expansion as a consequence of chemotaxis-driven RI. In the presence of chemotaxis, both the SL and DL expand simultaneously with RI (B, coloring for visual aid only), while no expansion is observed without chemotaxis (C).

(D–F) Time-lapse imaging of epiboly in live embryos (D) reveals that while SCs drift away from one another following CoMo treatment (E), the distance of SCs in C3aR-deficient embryos does not increase (F) during the process.

(G–I) Ex vivo study of tissue expansion using the intercalation assay (G). Cells in the DL separate as the isolated tissue undergoes expansion (H). This expansion is lost in tissues lacking C3aR (I).

(J) Quantification of tissue expansions as the difference in the final and initial distances relative to the initial distance of tracked cell pairs in silico, in vivo, and ex vivo. Error, SD; significance, **p ≤ 0.01, ***p < 0.001. See also Figure S7.

uniformly packed tissues could not produce such a gradient due to the lack of cell-free regions. The mechanism is less efficient with ubiquitous expression than with the segregated expression of the control case. Finally, a localized source of chemoattractant in the SL (Figure 8D, red) is unable to rescue local tissue thinning in silico. The chemotactic gradient in this setting does not only contain a radial component but also has a significant tangential component that is parallel to the SL-DL interface (Figure 8H). This gradient is attracting DCs from lateral regions and paradoxically leads to tissue thickening at the region of the active tissue.

To test these predictions, we measured the thickness of the BCR cross-section in stage 11 embryos after epiboly in conditions related to the model simulations (Figures 8I–8L, white bar marks tissue thickness). Control and inhibition measurements were performed in CoMo-and C3Mo-treated embryos (Figures 8I and 8J). Ubiquitous expression was achieved by C3a mRNA injection leading to C3a overexpression in all cells and RI as predicted (Figure 8K, see also Figures S2A–S2E). Localized C3 expression was achieved by grafting a piece of SL from a C3a mRNA-injected embryo (marked with RLDx in Figure 8L) into a C3Mo-treated host embryo. After healing, at stage 11 the tissue at the region of the graft was observed to be thicker than in either the control or the inhibited case indicating cell accumulation under the graft and confirming our prediction.

Quantification of tissue thicknesses shows the similarity of in silico predictions and in vivo validation (Figure 8M); while inhibition leads to significantly thicker tissues due to the lack of RI, ubiquitous overexpression shows no significant difference to the control. Note that, in silico, the phenotype is rescued with a 1.3-fold increase in expression levels, while the same expression levels result in a slightly but significantly thicker tissue (2.75 ± 0.08 cell diameters) than the control (2.3 ± 0.05 cell diameters). This shows that the ubiquitous expression pattern gives rise to a less efficient mechanism for thinning. A localized source of chemoattractant produces a significantly thicker tissue in the active region than the control, even surpassing the thickness of the passive, inhibited condition.

In summary, the counterintuitive predictions of the ubiquitous and localized sources and their experimental validation show that this chemotaxis-based morphogenetic mechanism is both

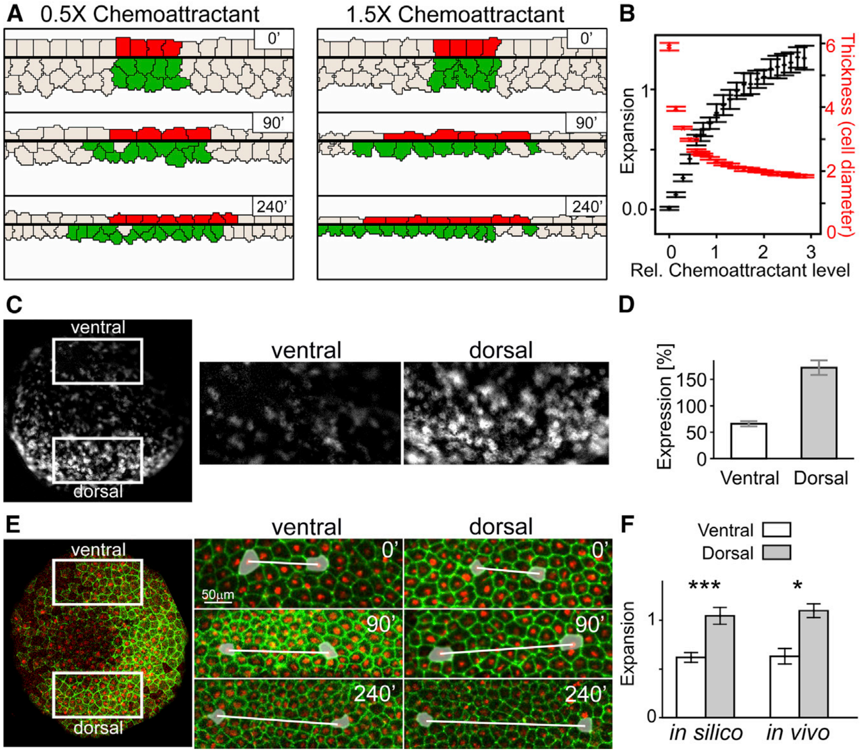

Figure 7. Dorsoventral Differences in Expansion Explained by Differential C3 Expression

(A) The extent of thinning and expansion depends on the level of chemoattractant in the SL in silico.

(B) Tissue expansion and thickness shown after 4 hr in simulations as a function of chemoattractant levels.

(C and D) Approximation of C3 levels in vivo using intensity levels from ISH of a stage 10 embryo reveals differential expression in the animal cap with a reduced level in the ventral region (66% compared with the whole-animal cap) and an increased level in the dorsal region (172%).

(E and F) Expansion in vivo in the ventral and dorsal areas of the animal pole. Expansion in the dorsal regions was significantly higher (n = 20) than in the ventral regions, as predicted by the model. Error bars in (B) and (F) show the SD; error bars in (D) show uncertainty of sampling. Significance ***$p < 0.001$, *$p < 0.05$.

robust and distinct from other chemotaxis processes where ubiquitous expression of the ligand impairs directional movement (e.g., chemotaxis of germ cells toward SDF-1; Doitsidou et al., 2002).

DISCUSSION

Here we present a molecular mechanism for RI based on short-range chemotaxis that can facilitate thinning and expansion of multilayered epithelia. In silico modeling of epiboly enabled us to predict intercalation dynamics, and sensitivity to chemotactic levels and localization. These predictions were tested in support of our hypothesis using a combination of in vivo, in vitro, and ex vivo assays.

We chose to study RI during *Xenopus* epiboly, where it was first described (Keller, 1980). We show that in this system, chemotaxis and consequent RI are driven and directed by the complement component C3 and its receptor C3aR. Although these components are best known for their role in the immune system, an increasing body of evidence suggests that these and other parts of the immune system are involved in functions unrelated to their immune function (Denny et al., 2013; Bénard et al., 2004; Hawksworth et al., 2014; Leslie and Mayor, 2013). C3a, together with C5a, has been shown to play a role in regeneration of a vast variety of tissues through promoting cell survival, proliferation, differentiation, and chemotaxis (Schraufstatter et al., 2015). During early development, the presence of C3 and its receptor has been reported in the *Xenopus* gastrula (McLin et al., 2008) and mouse neurula (Jeanes et al., 2015), however, their function in these contexts remained unknown. The function of C3 signaling has been identified in the migrating neural crest (NC), where it promotes cohesion of the migrating collective (Carmona-Fontaine et al., 2011). Importantly, C3 and C3aR are expressed in the same cells during NC migration, and therefore the mechanism of chemotaxis utilized by the NC differs from the one proposed here, where expression of C3 and its receptor is segregated (Carmona-Fontaine et al., 2011; Woods et al., 2014).

Separation of ligand and receptor has been reported in many chemotaxis processes, such as during the in vivo chemotaxis of primordial germ cells (PGCs) in zebrafish (Doitsidou et al., 2002) or the short-range chemotaxis of leukocyte trafficking through the vascular endothelium (Zabel et al., 2015). In such systems, the localization of the source determines the behavior, which can be tested under at least three extreme conditions. First, a complete inhibition of the source leads to the lack of directional migration, as observed for PGCs (Doitsidou et al., 2002) and in our cells in vitro (Figures 4C, 4E, and 4H). In vivo, the absence of chemoattractants led to the severe reduction of intercalation and of thinning (Figures 2 and 8J), as predicted in silico (Figures 8B and 8M). Second, if the chemoattractant is expressed in all tissues ubiquitously, the chemoattractant gradient is lost and chemotaxis is expected to fail, as seen for the PGCs (Doitsidou et al., 2002). However, ubiquitous expression in our system does not share the phenotype with the inhibited case (Figure 8K) and is not predicted to do so (Figure 8C). In this specific geometry, the relatively thin tissue is bound by the vast blastocoel cavity, which acts as a sink for the chemoattractant and gives rise to the gradient (Figure 8G inset). Such source-sink systems are known to produce robust and steep gradients (Majumdar et al., 2014) and have been described in vitro (Volpe et al., 2012; Scherber et al., 2012) and have been suggested in vivo (Yu et al., 2009; Boldajipour et al., 2008; Donà et al., 2013). Nevertheless, our model demonstrates that this mechanism is less efficient than the control, which could explain the segregation. Finally, an ectopically localized source is expected to misdirect chemotaxing cells. As our chemotaxis-based hypothesis

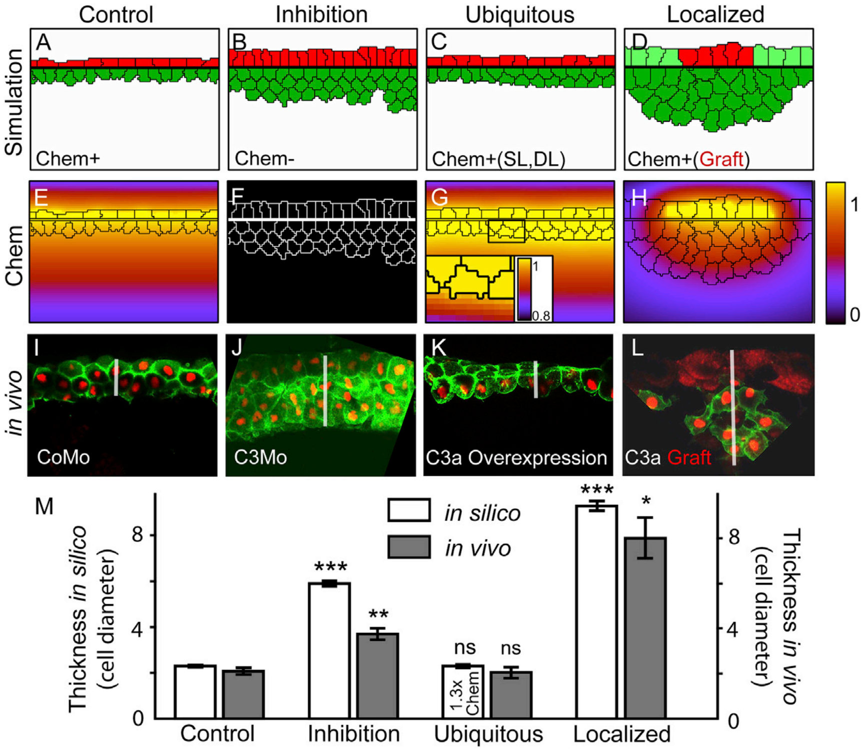

Figure 8. Localization of Chemoattractant Source Determines Radial Intercalation

(A–H) Simulated cell configurations and corresponding chemoattractant levels at the end of epiboly with different localizations of chemoattractant production: only in the SL (Control, A, E), nowhere (Inhibition, B, F), in every cell (Ubiquitous, C, G), and in a restricted region of the SL (Localized, D, H, producing region shown in red).

(I–L) Experimental validation of the predicted behavior showing sections of the blastocoel roof of stage 11 embryos. Red, nucleus; green, membrane; graft in (L) marked with cytosolic RLDx. White bar indicates tissue thickness.

(M) Quantification of tissue thickness, measured in units of cell diameter after normal epiboly (20 μm), for the four settings in silico and in vivo. Error, SD. Significances compared with relevant control: *p < 0.05, **p < 0.01, ***p < 0.001; ns, p > 0.05. 1.3× Chem denotes 1.3-fold overexpression in both DCs and SCs.

predicted, we found that introducing a local source of chemoattractant into a depleted embryo directs the cells toward this ectopic source to create a thick accumulation (Figure 8). Notably, this local re-establishment of the chemoattractant activity did not produce a local rescue. Taken together, our experiments of perturbed chemoattractant localization show that both our hypothesis and experimental setup behave as a chemotactic system.

Our results show that chemotaxis and DL intercalation are largely required for the expansion of the whole tissue occurring during epiboly, and that this expansion is independent of the rest of the embryo. Expansion of the non-intercalating SL is explained by our model through an effective friction between the DL and SL representing general cell adhesion or other independent mechanical interactions (Bergert et al., 2015). An alternative explanation for the SL-DL interaction with a similar expected result is that contact with the DCs could lower the basal surface tension of the SL in a C3/C3aR-dependent manner, allowing it to expand (Luu et al., 2011). As DL expansion in our model is driving SL expansion, the SL cannot expand beyond the DL. However, simulations with localized expression (Figure 8) reveal that the secreting region must be at least the same size as the receptive tissue; otherwise DCs outside the active region counteract expansion. While the boundary region of the SL and the neighboring mesoderm are beyond the scope of this study, we can contemplate that a yet unknown mechanism is at work to autonomously expand the SL beyond the DL. Such a mechanism is expected to act independently of C3 signaling, as its receptor is not expressed in the SL.

An apparently equivalent chemotaxis-based mechanism has been reported for the RI of the prechordal mesoderm (PCM) during *Xenopus* gastrulation (Damm and Winklbauer, 2011). These cells involute and subsequently migrate away from the blastopore against the expanding BCR. Cells of the PCM chemotax toward platelet-derived growth factor A (PDGF-A), produced by the nearby BCR, leading to the RI of the PCM, much like in the case of the expanding animal pole. Due to experimental limitations, expansion of the PCM is less accessible than the expansion analyzed in our system. It is tempting to speculate that the two processes share the same mechanism, and therefore any expansion resulting from the RI of the PCM may contribute to the expansion of the BCR, however this remains to be explored.

Our mechanism acts independently of the previously proposed adhesion-based mechanisms of epiboly and does not exclude them, as shown by the unaltered cell-cell adhesion and cell-fibronectin adhesion properties after blocking C3 signaling (Figures S3 and S4). The effect of C3 on DC protrusive activity is different from alternative adhesion-based hypotheses where a direct contact would be essential, e.g., DC protrusions promoted or stabilized on the surface of SCs, as supported by our observation that local C3 expression leads to accumulation of multiple layers of DCs around the active region, affecting even DCs that are not in direct contact with the SCs (Figure 8L). Nevertheless, our data do not exclude an essential role for fibronectin in epiboly, such as polarizing cells (Marsden and DeSimone, 2001; Petridou et al., 2013), preventing elevated tension in the animal cap (Petridou et al., 2013), regulating protrusions (Davidson et al., 2006), or even sequestering C3a to contribute to the gradient (Carmona-Fontaine et al., 2011). However, it is unlikely that these effects of fibronectin are sufficient to account for epiboly, as the matrix appears only hours after epiboly starts, and inhibition of cell-fibronectin binding is unable to affect the first half of epiboly (Marsden and DeSimone, 2001; Davidson et al., 2004). Therefore it is possible that C3 is required for the initiation and direction of RI and epibolic movements, and fibronectin is required for supporting and maintaining these movements during epiboly. Gastrulation is

disrupted by blocking either C3 signaling (Figure S1) or cell-fibronectin binding (Marsden and DeSimone, 2001; Petridou et al., 2013), showing the importance of epiboly for blastopore closure. Furthermore, epiboly also depends on the correct alignment of SC divisions through opposing apicobasal forces to keep the plane of division parallel to the SL (Woolner and Papalopulu, 2012). Upon disruption of these intracellular forces, SCs divide out of the epithelial plane and consequently epiboly is compromised (Woolner and Papalopulu, 2012). In-plane cell divisions have been shown to orient in order to relieve anisotropic tissue tension within the enveloping layer during zebrafish epiboly (Campinho et al., 2013). However, de-coupling of division orientation from tissue tension did not markedly hamper the progress of epiboly, although it led to abnormal fusion of the EVL cells (Campinho et al., 2013). While tissue organization in C3/C3aR-depleted embryos is affected, we observed no effect on the division of SCs in these embryos, making it unlikely that C3 signaling would be directly involved in the control of SC division.

Although the graded expression of E-cadherin required for the cell-cell adhesion-based mechanism is controversial (Song et al., 2013), there is strong evidence that the dynamics of E-cadherin is essential for epiboly in zebrafish (Babb and Marrs, 2004; Kane et al., 2005; Song et al., 2013; Shimizu et al., 2005; Arboleda-Estudillo et al., 2010). This dynamic cell adhesion is consistent with our model where DCs slide past the SL, similar to what has been proposed for the migration of prechordal plate mesendoderm, germ, and border cells (Ulrich et al., 2005; Kardash et al., 2010; Ulrich and Heisenberg, 2009; Cai et al., 2014), and also with recent data showing that guided cell migration in vivo requires dynamic filopodia (Meyen et al., 2015). Thus differential adhesion and chemotaxis could be acting in parallel. It would be interesting to know if an equivalent chemotactic mechanism of RI and epiboly based on the same C3a/C3aR molecules also operates in other animal models, such as zebrafish; however, the identification of the chemoattractant may be hampered by the presence of multiple C3 genes found in zebrafish (Forn-Cuní et al., 2014).

In summary, here we identify a molecular mechanism for RI based on short-range chemotaxis. We demonstrate that this mechanism is also involved in generating expansion during epiboly, both in the intercalating DL and non-intercalating SL. Chemotaxis in our particular study system is driven by the complement component C3, which has only been implicated in autocrine chemotaxis during development (Carmona-Fontaine et al., 2011). While the exact molecules may vary, this general mechanism of RI is likely to operate in other various epithelial morphogenetic events throughout developmental and pathological processes.

EXPERIMENTAL PROCEDURES

Embryology

Xenopus laevis embryos were obtained by in vitro fertilization and manipulated as previously described (Keller, 1978). Stages were determined according to Nieuwkoop and Faber (1967). Injections were performed in the animal blastomeres of 2- to 8-cell-stage embryos. Animal licenses were approved by the Home Office and University College London.

Staining and Imaging

The following probes were used: C3 (Carmona-Fontaine et al., 2011), Keratin (Kuriyama and Mayor, 2009), Xbra (Kuriyama and Mayor, 2009), Crescent (Ploper et al., 2011), and Wnt8 (Steventon et al., 2009). Histological sections and immune staining were performed as described elsewhere (Marsden and DeSimone, 2001). Western blot and SEM were executed as previously described (Carmona-Fontaine et al., 2011; Keller, 1980).

Quantifications

BCR thickness was measured as total tissue thickness in sections of fixed embryos (n = 25) at ten points per embryo. For ease of comparison with in silico data, thickness is expressed in units of cell diameters at the end of epiboly approximated to be 20 μm. The number of cell layers was counted at ten positions in each embryo, one section per embryo.

The direction of protrusions was estimated as the angle enclosed by the protrusion, the cell centroid, and a line drawn perpendicular to the line of the SL (Figure 5D, red angle). Angles range from 0° (pointing outward, toward the SL) to 180° (pointing inward, away from the SL).

Expansion was quantified by measuring the distance of selected cell nuclei at stage 11 (d_f). The selected cells were traced back to their ancestors at stage 9 by manual tracing, and the distance of the ancestors' nuclei (d_i) was measured. Expansion for each cell pair is calculated as $E = (d_f - d_i)/d_i$. The reported expansion of the cell layers is the average of all the measured cell pair expansions.

Expression levels of C3 for Figures 7C and 7D were measured using ImageJ. The ISH image was inverted, digitally cleaned of non-specific background (Subtract Background function in ImageJ), and contrast-enhanced. Gray levels were measured on the image in ten independent selections of the dorsal, the ventral, and the whole-animal cap regions. These technical repeats establish the uncertainty of the area selection and their SD is shown in Figure 7D as error bars.

Statistical Methods

Each experiment was repeated at least three times to ensure the reproducibility of the results. Due to the nature of *Xenopus laevis* in vitro fertilization, at least 200 embryos were gained for each experiment, which proved to be sufficient for establishing statistical significance in the results. For randomization purposes, different frogs were used in the experimental repeats. Simulations were repeated 30 times for all parameter sets. All data proved to be normally distributed therefore a standard one-sided Student's t test was used for assessing significances. All center values reported are averages; spreads are reported as SDs.

Computational Model

A cellular Potts model was implemented using the CompuCell3D platform (Swat et al., 2012). Cells in this model are represented as connected domains on a grid with the cell-free area represented as a special domain. Chemoattractant concentrations are represented on the same grid. Cell dynamics results from a series of attempts to expand the domains at randomly selected grid sites. Whether an expansion attempt is accepted or not depends on a set of rules, which thus determine cell dynamics. Cells are required to maintain an approximately constant volume. DC chemotaxis is implemented by favoring extensions that occur in the direction of the local chemoattractant gradient. Chemoattractant production is implemented by maintaining a constant level of chemoattractant at the sites of producing cells. The chemoattractant is allowed to diffuse and decay, giving rise to a gradient.

Simulations represent a single-cell-thick quasi-2D section of the BCR. SCs are initialized as a single layer of cells. To ensure epithelial integrity in the model, each SC is assigned a neighbor at the start. If the contact area with a neighbor falls below a threshold, the two neighbors are gently forced to move toward each other until their contact area is restored above the threshold. A thin immutable layer is introduced between the SL and DL to prevent any artificial grid effects or DC intercalation in between the SCs. The SCs are also required to maintain a minimal contact area with this layer at all times.

To implement coupling between the SL and DL, once a DC makes contact with the SL (that is, the immutable layer), an elastic connection is established between the DC and the SC immediately above it with a fixed probability. This connection may be broken if the DC loses contact with the immutable layer, or the connection reaches twice its original length, or with a given probability. Such connections have been demonstrated to describe tissue viscosity arising due to cell-cell contacts in keratinocytes (Czirók et al., 2013).

Division of SCs and DCs is implemented by dividing each cell through its midline once during the simulations. The time of division is determined by a cell-autonomous internal timer for each cell ensuring asynchronous cell divisions. Since cells do not grow, the volume of the two daughter cells is half of the volume of the mother cell. DCs divide along a randomly determined axis. The division plane of SCs is always perpendicular to the SL plane as described by experimental observations (Longo et al., 2004; Woolner and Papalopulu, 2012). After cell divisions, all neighbor relations for SCs and connections between DCs and SCs are re-established.

To achieve neutral lateral boundary conditions that allow expansion of the tissue and prevent it from collapsing, special lateral boundary conditions were implemented. Immutable anchor points are introduced 30 lattice sites from the lateral boundaries of the simulation area. Upon contact, SCs establish a neighbor connection with the anchor. All SCs that have their center of mass outside the anchor and are not connected to an anchor are considered outside the simulation area. In additional, if an SC is outside the anchor and all its neighbors are connected to the anchor, it is also considered outside the simulation. Connections of such cells are cut and their volume constraint is lifted to allow them to shrink and eventually be removed from the simulation. Similarly, DCs outside the line of the anchor without any connections lose their volume constraint, their ability to connect to any other SCs, and their ability to chemotax. However, if no DC is present in the area below the anchor point, a new DC is introduced in the simulation at that point.

To achieve mechanical equilibrium of the expanding tissue, a sufficiently high time resolution was used (Szabó et al., 2012).

Built-in routines were used for chemotaxis and secretion for the implementation. Epithelial integrity, cell divisions, DL-SL interaction, and boundary conditions were implemented as custom steppables.

For more information on the methods, see the Supplemental Information.

SUPPLEMENTAL INFORMATION

Supplemental Information includes Supplemental Experimental Procedures, seven figures, and seven movies and can be found with this article online at http://dx.doi.org/10.1016/j.devcel.2016.04.008.

AUTHOR CONTRIBUTIONS

Conceptualization, R.M.; Methodology, R.M. and A.S.; Software, A.S.; Formal Analysis, A.S., I.C., and R.M.; Investigation, I.C., S.O., S.M., R.K., and R.M.; Writing – Original Draft, A.S. and R.M.; Writing – Review & Editing, A.S., S.M., R.K., and R.M.; Visualization, A.S. and R.M.; Supervision, R.M.; Project Administration, R.M.; Funding Acquisition, A.S., S.M., and R.M.

ACKNOWLEDGMENTS

We thank C. Stern, J. Leslie, and M. Tada for comments on the manuscript, and J. Lambris for providing us with the C3a peptide and for encouraging us to explore the non-immunological functions of the complement. Fibronectin antibody was kindly provided by D. DeSimone. This study was supported by grants from the MRC (J000655 and M010465), BBSRC (M008517), and the Wellcome Trust (084247/Z/07/Z) to R.M. A.S. was supported by a Marie Curie Fellowship (329968), S.M. by a Wellcome Trust PhD studentship. The authors acknowledge the use of the UCL Legion High Performance Computing Facility (Legion@UCL), and associated support services, in the completion of this work.

Received: February 9, 2016
Revised: March 18, 2016
Accepted: April 8, 2016
Published: May 9, 2016

REFERENCES

Arboleda-Estudillo, Y., Krieg, M., Stühmer, J., Licata, N.A., Muller, D.J., and Heisenberg, C.-P. (2010). Movement directionality in collective migration of germ layer progenitors. Curr. Biol. *20*, 161–169.

Babb, S.G., and Marrs, J.A. (2004). E-cadherin regulates cell movements and tissue formation in early zebrafish embryos. Dev. Dyn. *230*, 263–277.

Bauer, D.V., Huang, S., and Moody, S.A. (1994). The cleavage stage origin of Spemann's Organizer: analysis of the movements of blastomere clones before and during gastrulation in *Xenopus*. Development *120*, 1179–1189.

Bénard, M., Gonzalez, B.J., Schouft, M.-T., Falluel-Morel, A., Vaudry, D., Chan, P., Vaudry, H., and Fontaine, M. (2004). Characterization of C3a and C5a receptors in rat cerebellar granule neurons during maturation. J. Biol. Chem. *279*, 43487–43496.

Bensch, R., Song, S., Ronneberger, O., and Driever, W. (2013). Non-directional radial intercalation dominates deep cell behavior during zebrafish epiboly. Biol. Open *2*, 845–854.

Bergert, M., Erzberger, A., Desai, R.A., Aspalter, I.M., Oates, A.C., Charras, G., Salbreux, G., and Paluch, E.K. (2015). Force transmission during adhesion-independent migration. Nat. Cell Biol. *17*, 524–529.

Boldajipour, B., Mahabaleshwar, H., Kardash, E., Reichman-Fried, M., Blaser, H., Minina, S., Wilson, D., Xu, Q., and Raz, E. (2008). Control of chemokine-guided cell migration by ligand sequestration. Cell *132*, 463–473.

Cai, D., Chen, S.-C., Prasad, M., He, L., Wang, X., Choesmel-Cadamuro, V., Sawyer, J.K., Danuser, G., and Montell, D.J. (2014). Mechanical feedback through E-Cadherin promotes direction sensing during collective cell migration. Cell *157*, 1146–1159.

Campinho, P., Behrndt, M., Ranft, J., Risler, T., Minc, N., and Heisenberg, C.-P. (2013). Tension-oriented cell divisions limit anisotropic tissue tension in epithelial spreading during zebrafish epiboly. Nat. Cell Biol. *15*, 1405–1414.

Carmona-Fontaine, C., Theveneau, E., Tzekou, A., Tada, M., Woods, M.L., Page, K.M., Parsons, M., Lambris, J.D., and Mayor, R. (2011). Complement fragment C3a controls mutual cell attraction during collective cell migration. Dev. Cell *21*, 1026–1037.

Chen, P., Johnson, J.E., Zoghbi, H.Y., and Segil, N. (2002). The role of Math1 in inner ear development: uncoupling the establishment of the sensory primordium from hair cell fate determination. Development *129*, 2495–2505.

Clark, I.B.N., Muha, V., Klingseisen, A., Leptin, M., and Müller, H.-A.J. (2011). Fibroblast growth factor signalling controls successive cell behaviours during mesoderm layer formation in *Drosophila*. Development *138*, 2705–2715.

Czirók, A., Varga, K., Méhes, E., and Szabó, A. (2013). Collective cell streams in epithelial monolayers depend on cell adhesion. New J. Phys. *15*, 075006.

Damm, E.W., and Winklbauer, R. (2011). PDGF-A controls mesoderm cell orientation and radial intercalation during *Xenopus* gastrulation. Development *138*, 565–575.

Davidson, L.A., Keller, R., and DeSimone, D.W. (2004). Assembly and remodeling of the fibrillar fibronectin extracellular matrix during gastrulation and neurulation in *Xenopus laevis*. Dev. Dyn. *231*, 888–895.

Davidson, L.A., Marsden, M., Keller, R., and DeSimone, D.W. (2006). Integrin $\alpha5\beta1$ and fibronectin regulate polarized cell protrusions required for *Xenopus* convergence and extension. Curr. Biol. *16*, 833–844.

Denny, K.J., Coulthard, L.G., Jeanes, A., Lisgo, S., Simmons, D.G., Callaway, L.K., Wlodarczyk, B., Finnell, R.H., Woodruff, T.M., and Taylor, S.M. (2013). C5a receptor signaling prevents folate deficiency-induced neural tube defects in mice. J. Immunol. *190*, 3493–3499.

Doitsidou, M., Reichman-Fried, M., Stebler, J., Köprunner, M., Dörries, J., Meyer, D., Esguerra, C.V., Leung, T., and Raz, E. (2002). Guidance of primordial germ cell migration by the chemokine SDF-1. Cell *111*, 647–659.

Donà, E., Barry, J.D., Valentin, G., Quirin, C., Khmelinskii, A., Kunze, A., Durdu, S., Newton, L.R., Fernandez-Minan, A., Huber, W., et al. (2013). Directional tissue migration through a self-generated chemokine gradient. Nature *503*, 285–289.

Forn-Cuní, G., Reis, E.S., Dios, S., Posada, D., Lambris, J.D., Figueras, A., and Novoa, B. (2014). The evolution and appearance of C3 duplications in fish originate an exclusive teleost c3 gene form with anti-inflammatory activity. PLoS One *9*, e99673.

Graner, F., and Glazier, J.A. (1992). Simulation of biological cell sorting using a two-dimensional extended Potts model. Phys. Rev. Lett. *69*, 2013–2016.

Hawksworth, O.A., Coulthard, L.G., Taylor, S.M., Wolvetang, E.J., and Woodruff, T.M. (2014). Brief report: complement C5a promotes human embryonic stem cell pluripotency in the absence of FGF2. Stem Cells *32*, 3278–3284.

Honczarenko, M., Ratajczak, M.Z., Nicholson-Weller, A., and Silberstein, L.E. (2005). Complement C3a enhances CXCL12 (SDF-1)-mediated chemotaxis of bone marrow hematopoietic cells independently of C3a receptor. J. Immunol. *175*, 3698–3706.

Höpker, V.H., Shewan, D., Tessier-Lavigne, M., Poo, M., and Holt, C. (1999). Growth-cone attraction to netrin-1 is converted to repulsion by laminin-1. Nature *401*, 69–73.

Jeanes, A., Coulthard, L.G., Mantovani, S., Markham, K., and Woodruff, T.M. (2015). Co-ordinated expression of innate immune molecules during mouse neurulation. Mol. Immunol. *68*, 253–260.

Kane, D.A., McFarland, K.N., and Warga, R.M. (2005). Mutations in half baked/E-cadherin block cell behaviors that are necessary for teleost epiboly. Development *132*, 1105–1116.

Kardash, E., Reichman-Fried, M., Maître, J., Boldajipour, B., Papusheva, E., Messerschmidt, E.-M., Heisenberg, C.-P., and Raz, E. (2010). A role for Rho GTPases and cell-cell adhesion in single-cell motility in vivo. Nat. Cell Biol. *12*, 11–47.

Kee, N., Wilson, N., De Vries, M., Bradford, D., Key, B., and Cooper, H.M. (2008). Neogenin and RGMa control neural tube closure and neuroepithelial morphology by regulating cell polarity. J. Neurosci. *28*, 12643–12653.

Keller, R.E. (1978). Time-lapse cinemicrographic analysis of superficial cell behavior during and prior to gastrulation in *Xenopus laevis*. J. Morphol. *157*, 223–247.

Keller, R.E. (1980). The cellular basis of epiboly: an SEM study of deep-cell rearrangement during gastrulation in *Xenopus laevis*. J. Embryol. Exp. Morphol. *60*, 201–234.

Kishimoto, Y., Murate, M., and Sugiyama, T. (1996). Hydra regeneration from recombined ectodermal and endodermal tissue. I. Epibolic ectodermal spreading is driven by cell intercalation. J. Cell Sci. *109* (Pt 4), 763–772.

Kuriyama, S., and Mayor, R. (2009). A role for Syndecan-4 in neural induction involving ERK- and PKC-dependent pathways. Development *136*, 575–584.

Leslie, J.D., and Mayor, R. (2013). Complement in animal development: unexpected roles of a highly conserved pathway. Semin. Immunol. *25*, 39–46.

Longo, D., Peirce, S.M., Skalak, T.C., Davidson, L.A., Marsden, M., Dzamba, B., and DeSimone, D.W. (2004). Multicellular computer simulation of morphogenesis: blastocoel roof thinning and matrix assembly in *Xenopus laevis*. Dev. Biol. *271*, 210–222.

Luu, O., David, R., Ninomiya, H., and Winklbauer, R. (2011). Large-scale mechanical properties of *Xenopus* embryonic epithelium. Proc. Natl. Acad. Sci. USA *108*, 4000–4005.

Majumdar, R., Sixt, M., and Parent, C.A. (2014). New paradigms in the establishment and maintenance of gradients during directed cell migration. Curr. Opin. Cell Biol. *30*, 33–40.

Málaga-Trillo, E., Solis, G.P., Schrock, Y., Geiss, C., Luncz, L., Thomanetz, V., and Stuermer, C.A.O. (2009). Regulation of embryonic cell adhesion by the prion protein. PLoS Biol. *7*, e55.

Marsden, M., and DeSimone, D.W. (2001). Regulation of cell polarity, radial intercalation and epiboly in *Xenopus*: novel roles for integrin and fibronectin. Development *128*, 3635–3647.

McLin, V.A., Hu, C.-H., Shah, R., and Jamrich, M. (2008). Expression of complement components coincides with early patterning and organogenesis in *Xenopus laevis*. Int. J. Dev. Biol. *52*, 1123–1133.

Merks, R.M.H., Newman, S.A., and Glazier, J.A. (2004). Cell-oriented modeling of in vitro capillary development. Lect. Notes Comput. Sci. *3305*, 425–434.

Meyen, D., Tarbashevich, K., Banisch, T.U., Wittwer, C., Reichman-Fried, M., Maugis, B., Grimaldi, C., Messerschmidt, E.-M., and Raz, E. (2015). Dynamic filopodia are required for chemokine-dependent intracellular polarization during guided cell migration in vivo. Elife *4*, 1–25.

Nieuwkoop, P., and Faber, J. (1967). Normal Table of *Xenopus Laevis* (Amsterdam: North-Holland).

Petridou, N.I., Stylianou, P., and Skourides, P.A. (2013). A dominant-negative provides new insights into FAK regulation and function in early embryonic morphogenesis. Development *140*, 4266–4276.

Ploper, D., Lee, H.X., and De Robertis, E.M. (2011). Dorsal–ventral patterning: crescent is a dorsally secreted Frizzled-related protein that competitively inhibits Tolloid proteases. Dev. Biol. *352*, 317–328.

Ricklin, D., Hajishengallis, G., Yang, K., and Lambris, J.D. (2010). Complement: a key system for immune surveillance and homeostasis. Nat. Immunol. *11*, 785–797.

Scarpa, E., Szabó, A., Bibonne, A., Theveneau, E., Parsons, M., and Mayor, R. (2015). Cadherin switch during EMT in neural crest cells leads to contact inhibition of locomotion via repolarization of forces. Dev. Cell *34*, 421–434.

Schepis, A., Sepich, D., and Nelson, W.J. (2012). αE-catenin regulates cell-cell adhesion and membrane blebbing during zebrafish epiboly. Development *139*, 537–546.

Scherber, C., Aranyosi, A.J., Kulemann, B., Thayer, S.P., Toner, M., Iliopoulos, O., and Irimia, D. (2012). Epithelial cell guidance by self-generated EGF gradients. Integr. Biol. *4*, 259.

Schraufstatter, I.U., Khaldoyanidi, S.K., and DiScipio, R.G. (2015). Complement activation in the context of stem cells and tissue repair. World J. Stem Cells *7*, 1090.

Shimizu, T., Yabe, T., Muraoka, O., Yonemura, S., Aramaki, S., Hatta, K., Bae, Y.-K., Nojima, H., and Hibi, M. (2005). E-cadherin is required for gastrulation cell movements in zebrafish. Mech. Dev. *122*, 747–763.

Song, S., Eckerle, S., Onichtchouk, D., Marrs, J.A., Nitschke, R., and Driever, W. (2013). Pou5f1-dependent EGF expression controls E-cadherin endocytosis, cell adhesion, and zebrafish epiboly movements. Dev. Cell *24*, 486–501.

Steventon, B., Araya, C., Linker, C., Kuriyama, S., and Mayor, R. (2009). Differential requirements of BMP and Wnt signalling during gastrulation and neurulation define two steps in neural crest induction. Development *136*, 771–779.

Sugrue, S.P., and Hay, E.D. (1981). Response of basal epithelial cell surface and cytoskeleton to solubilized extracellular matrix molecules. J. Cell Biol. *91*, 45–54.

Swat, M.H., Thomas, G.L., Belmonte, J.M., Shirinifard, A., Hmeljak, D., and Glazier, J.A. (2012). Multi-scale modeling of tissues using compu cell 3D. In Computational Methods in Cell Biology, A.R. Asthagiri and A.P. Arkin, eds. (Academic Press), pp. 325–366.

Szabó, A., Varga, K., Garay, T., Hegedűs, B., and Czirók, A. (2012). Invasion from a cell aggregate–the roles of active cell motion and mechanical equilibrium. Phys. Biol. *9*, 016010.

Ulrich, F., and Heisenberg, C.-P. (2009). Trafficking and cell migration. Traffic *10*, 811–818.

Ulrich, F., Krieg, M., Schötz, E.-M., Link, V., Castanon, I., Schnabel, V., Taubenberger, A., Mueller, D., Puech, P.-H., and Heisenberg, C.-P. (2005). Wnt11 functions in gastrulation by controlling cell cohesion through Rab5c and E-cadherin. Dev. Cell *9*, 555–564.

Volpe, S., Cameroni, E., Moepps, B., Thelen, S., Apuzzo, T., and Thelen, M. (2012). CCR2 acts as scavenger for CCL2 during monocyte chemotaxis. PLoS One *7*, e37208.

Warga, R.M., and Kimmel, C.B. (1990). Cell movements during epiboly and gastrulation in zebrafish. Development *108*, 569–580.

Woods, M.L., Carmona-Fontaine, C., Barnes, C.P., Couzin, I.D., Mayor, R., and Page, K.M. (2014). Directional collective cell migration emerges as a property of cell interactions. PLoS One *9*, e104969.

Woolner, S., and Papalopulu, N. (2012). Spindle position in symmetric cell divisions during epiboly is controlled by opposing and dynamic apicobasal forces. Dev. Cell *22*, 775–787.

Yamada, M., Udagawa, J., Matsumoto, A., Hashimoto, R., Hatta, T., Nishita, M., Minami, Y., and Otani, H. (2010). Ror2 is required for midgut elongation during mouse development. Dev. Dyn. *239*, 941–953.

Yen, W.W., Williams, M., Periasamy, A., Conaway, M., Burdsal, C., Keller, R.E., Lu, X., and Sutherland, A. (2009). PTK7 is essential for polarized cell motility and convergent extension during mouse gastrulation. Development *136*, 2039–2048.

Yu, S.R., Burkhardt, M., Nowak, M., Ries, J., Petrášek, Z., Scholpp, S., Schwille, P., and Brand, M. (2009). Fgf8 morphogen gradient forms by a source-sink mechanism with freely diffusing molecules. Nature *461*, 533–536.

Zabel, B.A., Rott, A., and Butcher, E.C. (2015). Leukocyte chemoattractant receptors in human disease pathogenesis. Annu. Rev. Pathol. *10*, 51–81.

Developmental Cell, Volume *37*

Supplemental Information

The Molecular Basis of Radial Intercalation during Tissue Spreading in Early Development

András Szabó, Isidoro Cobo, Sharif Omara, Sophie McLachlan, Ray Keller, and Roberto Mayor

Inventory of Supplemental Information

Figure S1, related to Figure 2. C3aR inhibition affects blastopore closure.
Figure S2, related to Figure 2. Complement component C3 is required for normal Xenopus epiboly.
Figure S3, related to Figure 2. C3aR inhibition does not affect cell-to-cell adhesion.
Figure S4, related to Figure 2. Fibronectin and cell-fibronectin adhesion are unaffected by C3aR inhibition.
Figure S5, related to Figure 2. Ectoderm and mesoderm specification is not affected by C3aR inhibition.
Figure S6, related to Figure 3. Ectoderm and mesoderm specification is not affected by C3aR inhibition.
Figure S7, related to Figure 6. Model behavior after changing the number of cell layers.
Movie S1. Ex vivo assay of radial intercalation.
Movie S2. Computational model simulation of radial intercalation.
Movie S3. In vitro chemotaxis assay using deep and superficial layer explants.
Movie S4. In vitro chemotaxis assay using deep layer explants and purified C3a.
Movie S5. Ex vivo assay showing protrusive activity.
Movie S6. In vivo expansion of the blastocoel roof during epiboly.
Movie S7. Computational model simulation with altered chemoattractant levels.
Supplemental Experimental Procedures
Supplemental References

Supplemental Figures

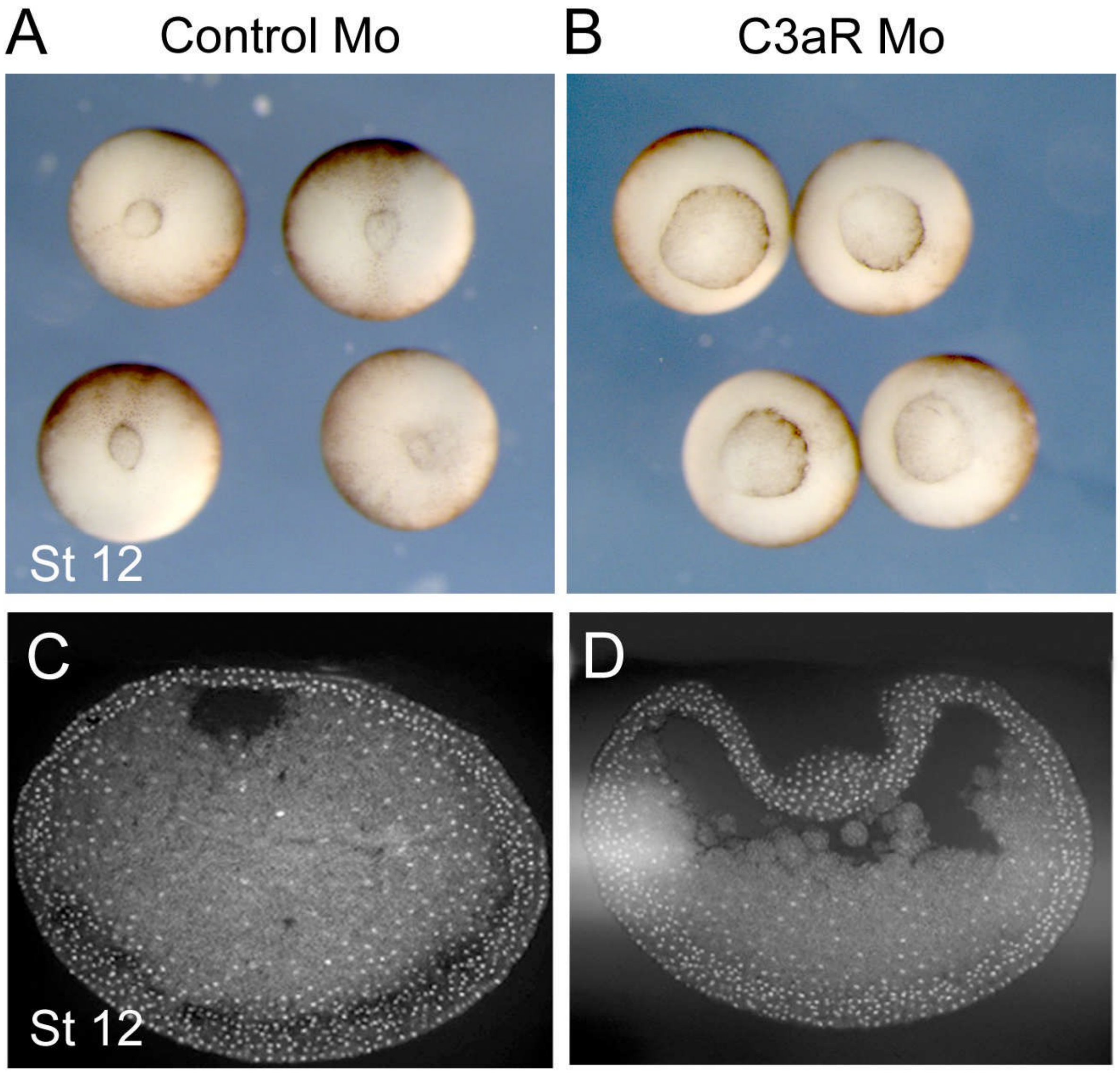

Figure S1. C3aR inhibition affects blastopore closure. Related to Figure 2

Blastopore of representative control (A, CoMo injected) and morphant (B, C3aRMo injected) embryos at stage 12. C3aR inhibition leads to failure of blastopore closure in 68% of the observed n=120 embryos. Internal structure of embryos shown on sections of whole control (C, CoMo) and morphant (D, C3aRMo) embryos.

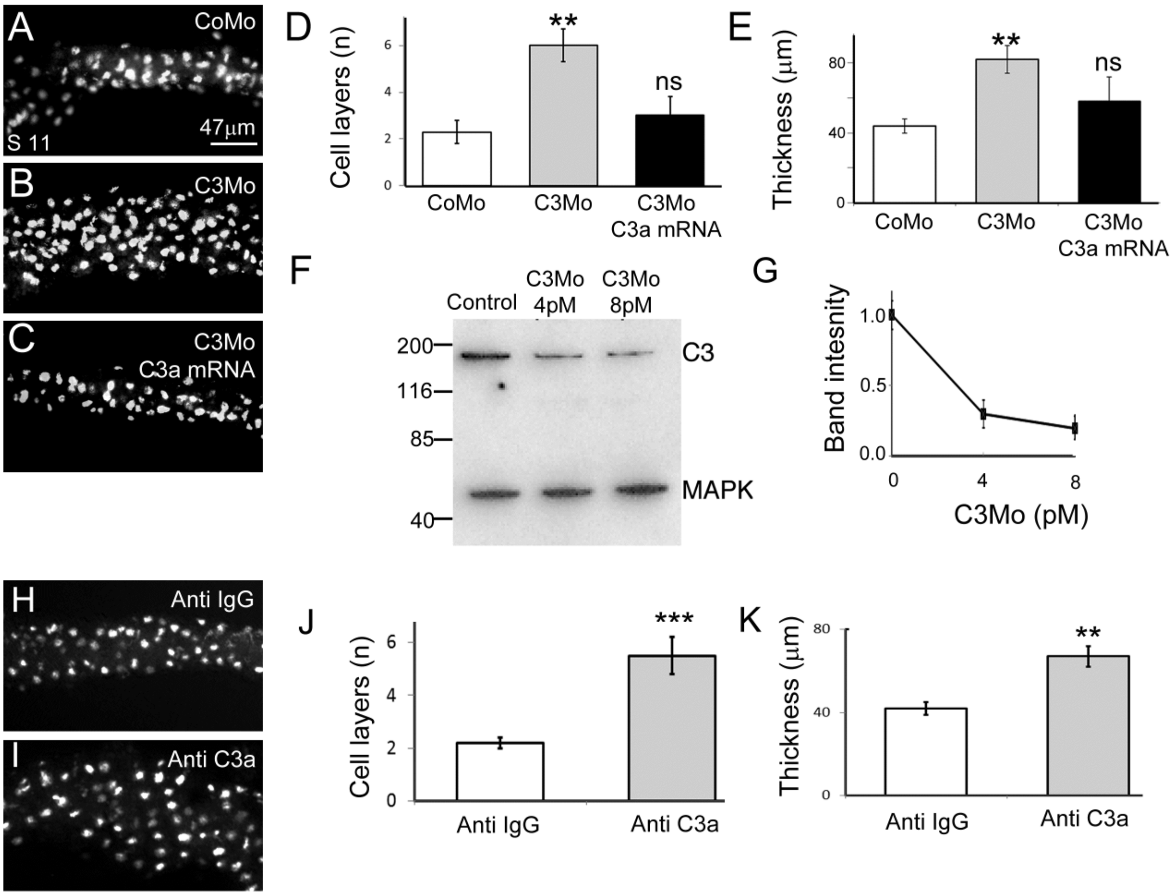

Figure S2. Complement component C3 is required for normal Xenopus epiboly. Related to Figure 2

Sections of the BCR of stage 11 embryos treated with CoMo (A), C3Mo (B), and C3Mo co-injected with rescue C3a mRNA that does not hybridize the C3Mo (C). The number of cell layers (D, n=60 embryos, p<0.01) and tissue thickness (E, n=60 embryos, p<0.01) are both significantly increased in C3 inhibited embryos compared to those in CoMo injected embryos. This effect is rescued by co-injecting C3Mo and C3a mRNA, in which case the difference in number of cell layers and tissue thickness is not significant when compared to those in CoMo injected embryos. This rescue shows the specificity of C3Mo. Western blot of CoMo and C3Mo injected SLs shows the dose-dependent inhibitory effect of the morpholino (F, G). All inhibitory experiments were carried out using 4pM Mo concentrations. (H, I) Sections of BCR of stage 11 embryos injected in the blastocoel cavity with pre-immune anti IgG antibody (H) or a blocking anti C3a antibody (I), previously characterized (Carmona-Fontaine et al., 2011). The number of cell layers (J, n=30 embryos, p<0.005) and tissue thickness (K, n=35 embryos, p<0.01) are both increased in embryos injected with the C3a antibody, but not with the control IgG. Error bars on D, E, G, J and K represent standard deviation.

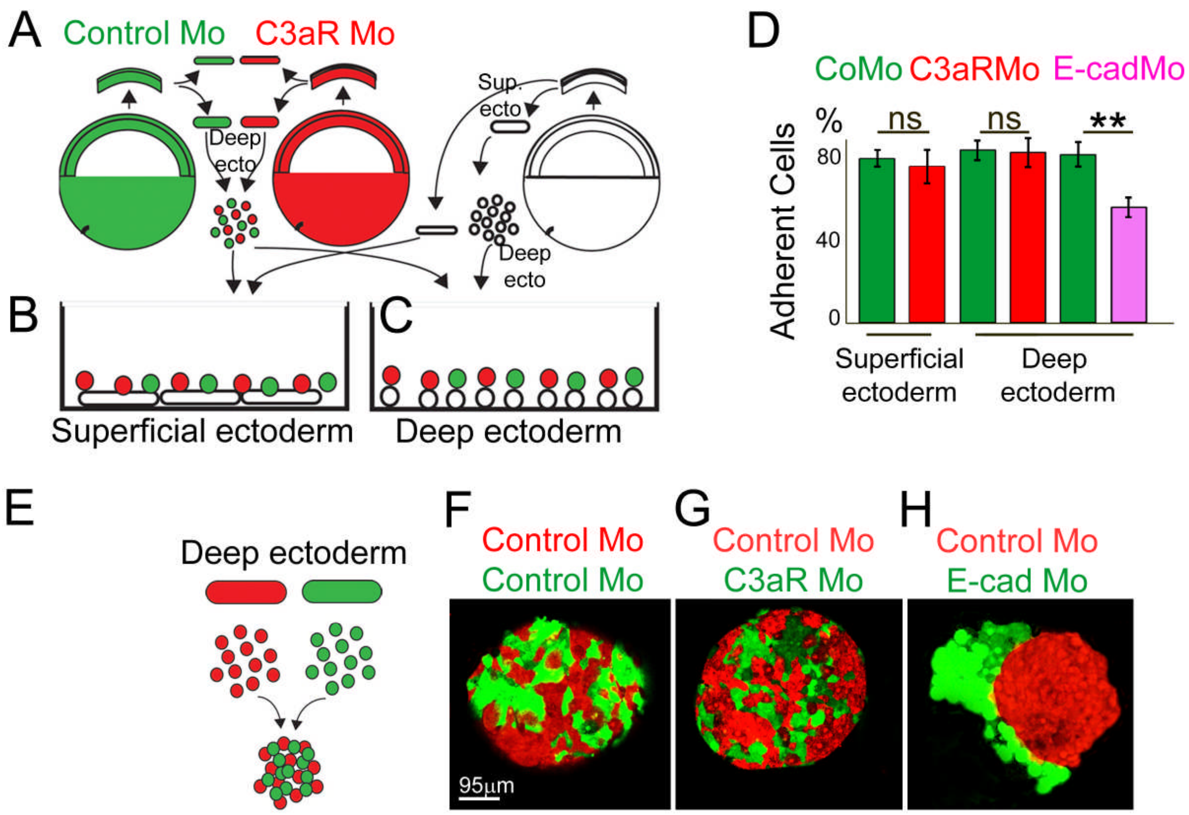

Figure S3. C3aR inhibition does not affect cell-to-cell adhesion. Related to Figure 2

(A-C) Deep ectoderm of stage 10 embryos injected with CoMo (green) or C3aRMo (red) were explanted onto a layer of superficial (B) or deep (C) ectoderm of untreated embryos (white, unlabeled). For positive control, deep ectoderm from CoMo and E-Cadherin Mo (E-cadMo) injected embryos were explanted on untreated deep ectoderm (not shown). After 2h the cultures were washed to remove detached cells and the percentage of variously treated cells remaining on superficial or deep ectoderm was quantified (D, n=80 experiments). No significant difference was observed in the attachment of CoMo and C3aR inhibited cells, however, the difference between control and E-Cadherin inhibited cells is significant ($p<0.01$, error bars: standard deviation).

(E-H) Cell sorting assays of deep ectodermal cells from two different stage 9 embryos were performed on agarose covered dishes. No adhesion difference was observed between cells explanted from CoMo and CoMo (F), or CoMo and C3aR inhibited (G) embryos, while cells originated from CoMo and E-Cadherin embryos clearly sorted (H).

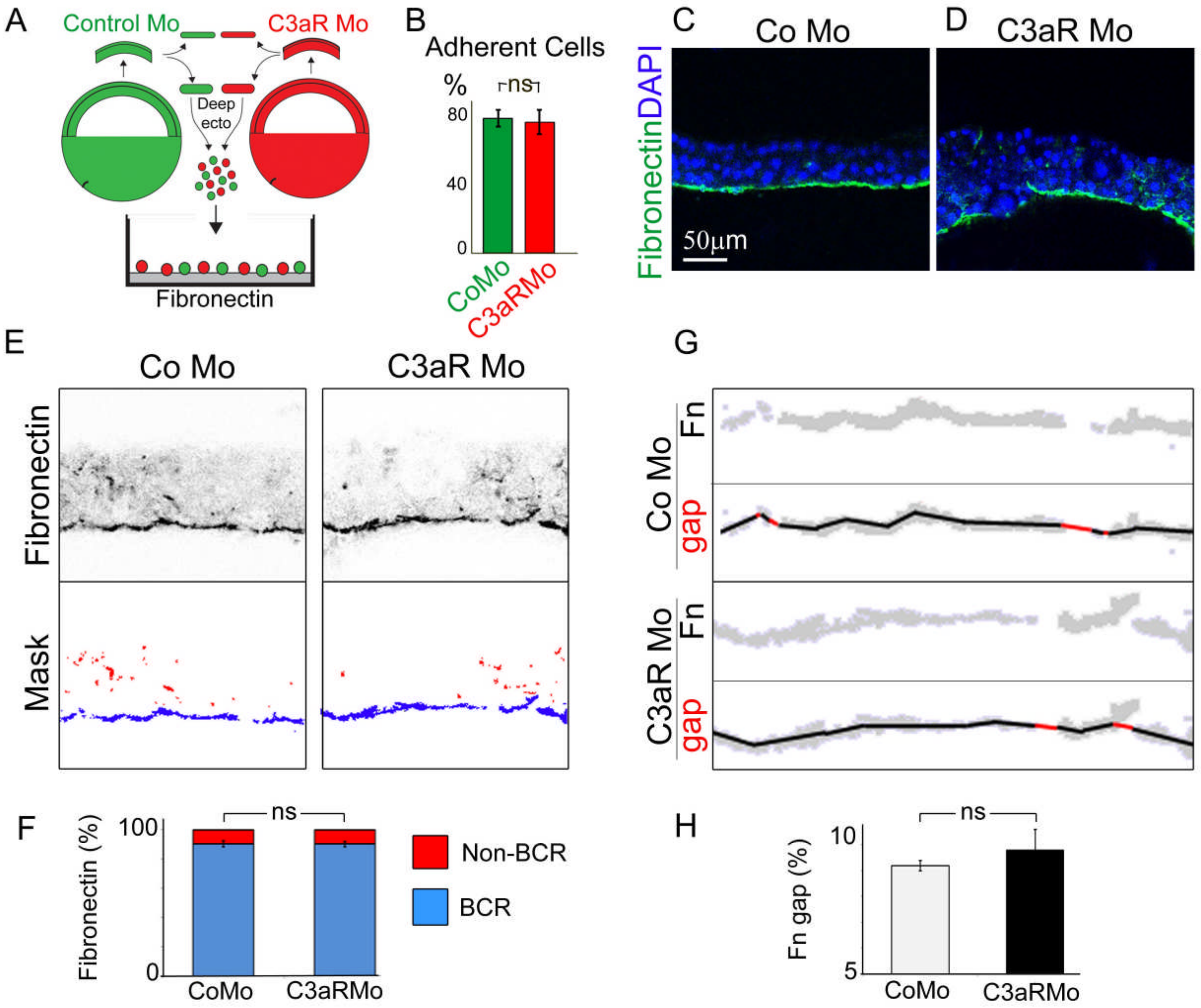

Figure S4. Fibronectin and cell-fibronectin adhesion are unaffected by C3aR inhibition. Related to Figure 2

(A-B) Deep ectodermal cells from CoMo (green) and C3aRMo treated (red) embryos at stage 10 were explanted, mixed, and plated on fibronectin substrate. After 2h of culture the cells were washed. (B) The percentage of adherent cells after the wash did not show significant difference between control and C3aR inhibited cells (n=30 experiments, error bars: standard deviation).

(C-H) Fibronectin accumulation at the BCR in vivo is unaffected by C3aR inhibition, as shown by immunostained sections of the BCR from stage 10 embryos (C-D). Most importantly, fibronectin is accumulated at the edge of the BCR despite the heavily disrupted tissue architecture. Note the occasional slight ectopic expression and discontinuity in the fibronectin in both CoMo (C, middle and right side) and C3aRMo (D) treated embryos.

Quantifications of fibronectin immunofluorescence intensities at the blastocoel roof (BCR, blue) and within the tissue (Non-BCR, red) show no significant difference between the control (CoMo) or morphant (C3aRMo) embryos (F). Continuity of the fibronectin layer at the BCR was assessed by measuring the length of BCR segments without fibronectin (gaps; G, red). This is then related to the total length of the BCR (red and black lines). No significant difference was found between control (CoMo) and treated (C3aRMo) embryos (H). Error bars: standard error of the mean.

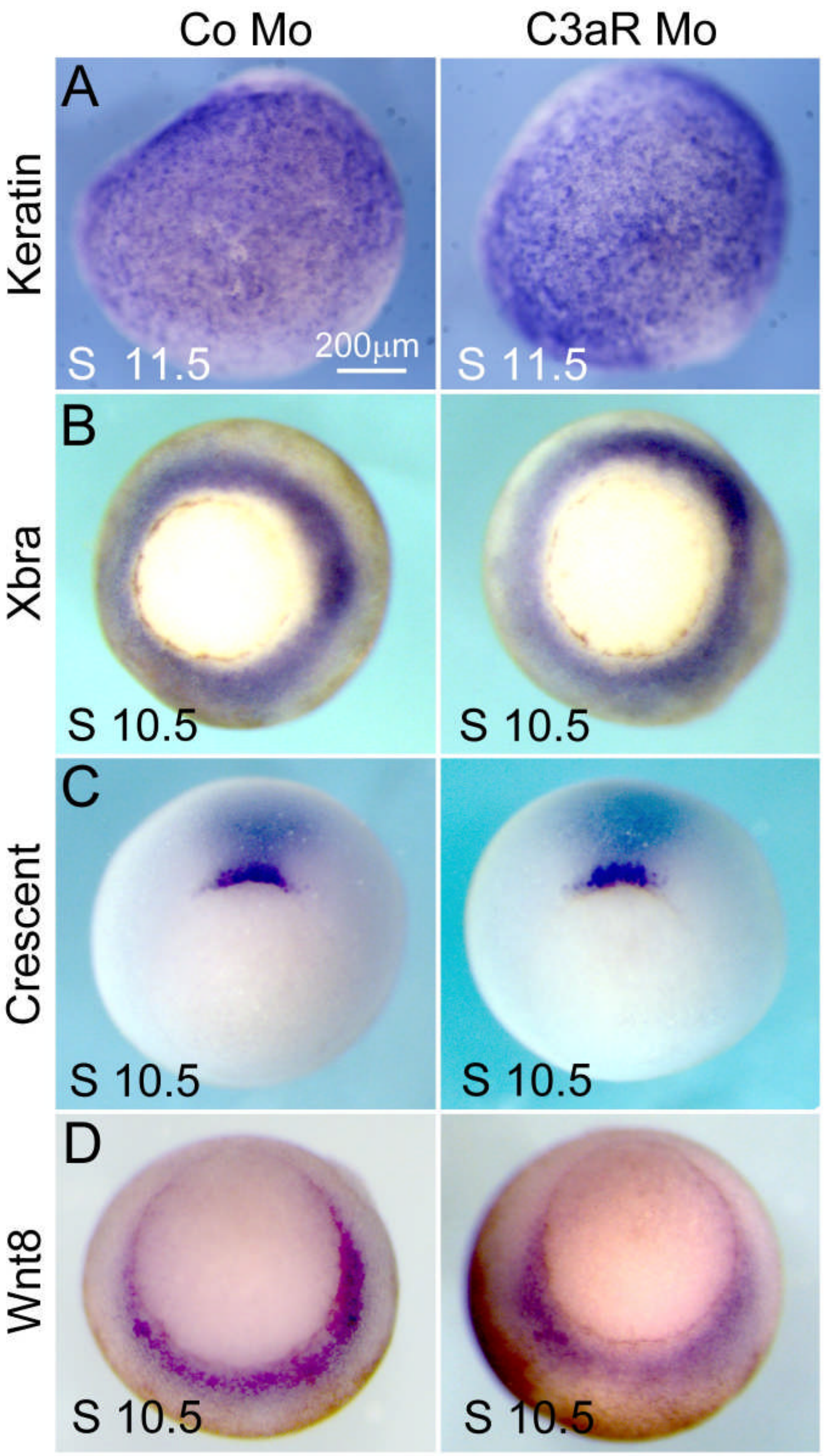

Figure S5. Ectoderm and mesoderm specification is not affected by C3aR inhibition. Related to Figure 2

(A) Keratin expression in the animal pole at stage 11.5 is normal in C3aRMo injected embryos, indicating the normal specification of the ectoderm (ISH, animal view).

(B-D) Mesodermal markers Brachyury (Xbra), Crescent, and Wnt8 at stage 10.5 are unaffected by C3aR inhibition (ISH, vegetal view).

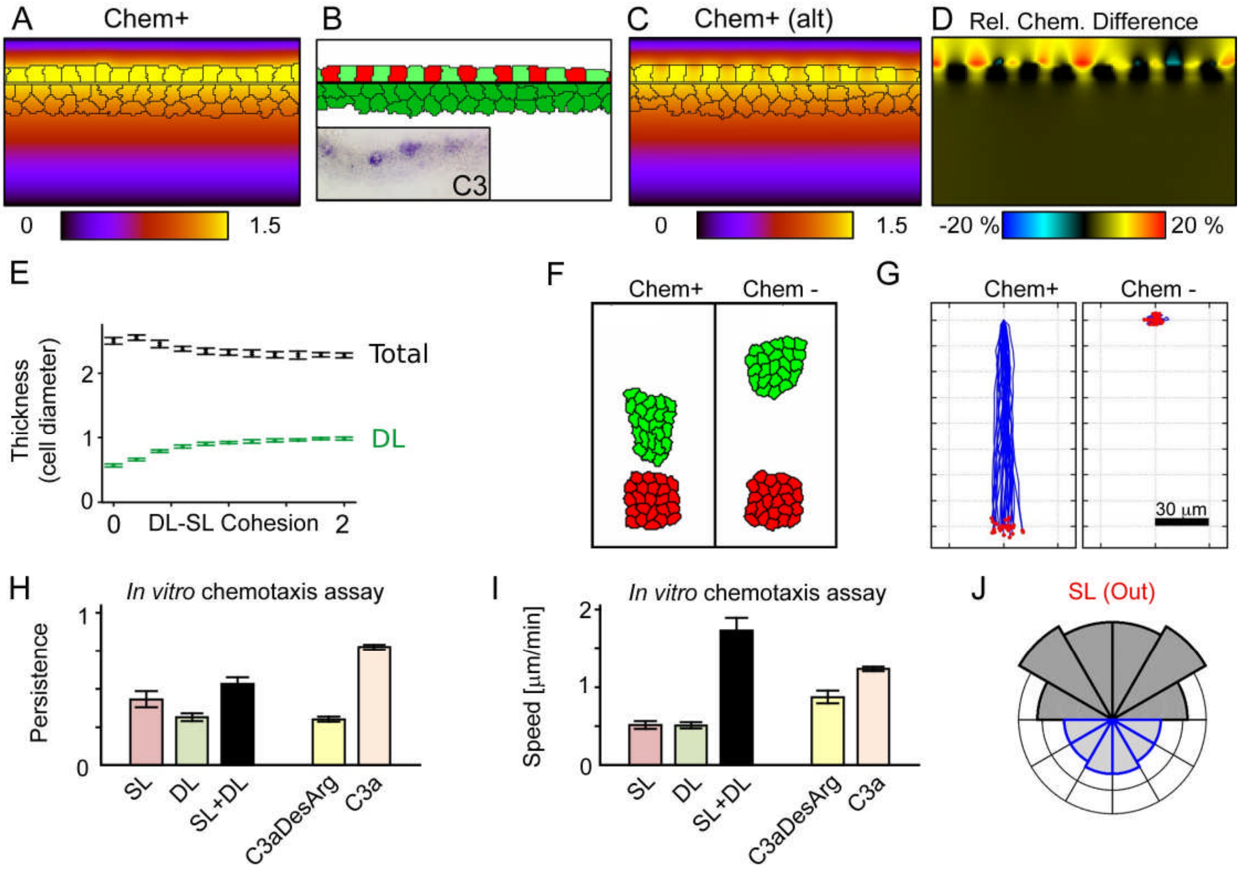

Figure S6. Computational model behavior. Related to Figure 3

(A-D) Alternating chemoattractant expression does not affect the chemoattractant gradient in the simulations. (A) Emergent chemoattractant field with overlaid cell outlines in a simulation where all SCs secrete. (B) Configurations using alternative chemoattractant expression inspired by the patterned ISH signal observed in animal pole sections (inset). (C) Chemoattractant field with overlaid cell outlines in the simulation shown in (B). (D) The point-by-point relative difference of the uniform (c_u, shown on A) and alternating (c_a, shown on C) chemoattractant fields defined as: $(c_u - c_a)/c_u$, diminishes further away from the SL where the difference is less than 10% homogeneously.

(E) Thickness of the whole BCR tissue (Total) and the DL as the function of effective friction between the superficial and deep layers (DL-SL Cohesion). Without cohesion the DL thins out, and as the friction is increased, DL thickness reaches a plateau within less than double of the minimal value. Meanwhile the total tissue thickness is largely unaffected. All simulations in the study are run with cohesion parameter = 1, the smallest value where the DL thickness starts to reach the plateau. Error bars represent standard deviation, n=30 simulations for each parameter set.

(F) Chemotaxis assay in silico showing chemotaxis of a DL explant (green) towards a cluster of SL explant (red) with (Chem+) and without (Chem-) chemotaxis. Explants are simulated on a 2D substrate.

(G) Trajectories of DL explants in silico with and without chemotaxis.

(H-I) Persistence (H) and speed (I) of clusters in the in vitro chemotaxis assay shown in Figure 4. Error bars: standard error of the mean.

(J) Distribution of protrusion directions of in vivo DCs that are completely surrounded by other cells, quantified from SEM images of fixed and fractured embryos (see main Figures 5A-D). This measurement excludes any potential bias towards the SL due to the lack of cell neighbors towards the blastocoel at the very bottom of the DL. This restricted distribution still shows a significant bias towards the SL, similar to Figure 5E.

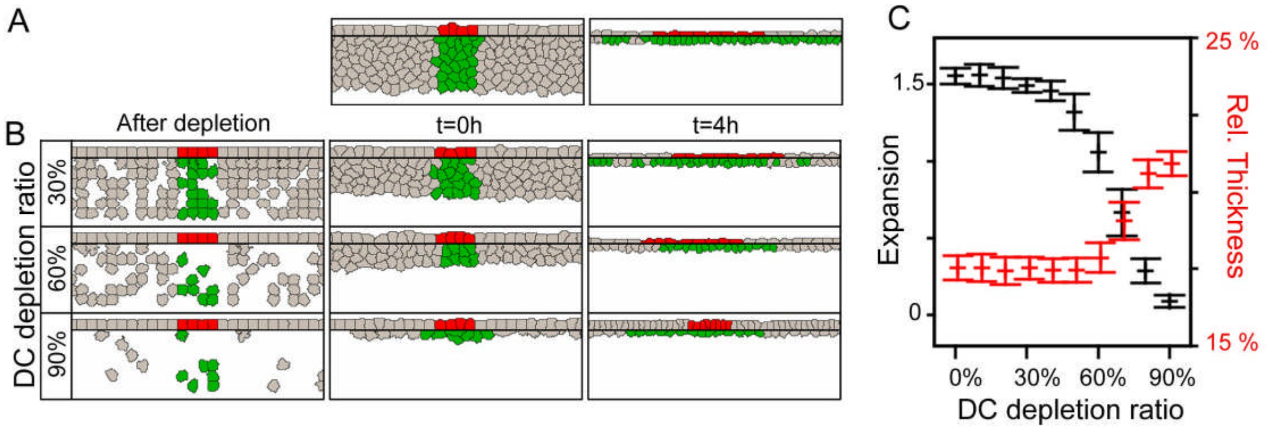

Figure S7. Model behavior after changing the number of cell layers. Related to Figure 6

(A) Configurations from a sample simulation run with 6 layers of DCs showing that the model reproduces epiboly in systems with more than 2 layers of DCs, as in zebrafish.

(B) Simulations initiated with 6 layers of DCs, after which a given percentage of the DC population (DC depletion ratio) is removed. The resultant holes are filled by the DCs condensing in the vicinity of the SL. The proposed chemotaxis-based mechanism in simulations with a large number of DCs produces effective expansion of the tissue. Expansion is hampered only when the number of DC layers falls below two.

(C) SL expansion and relative tissue thickness in simulations run with different ratio of DCs depleted from the initial 6 layers of DCs (see panels I and J). Thickness is relative to the initial tissue thickness (before depletion). Even when the majority of the DCs are depleted from the simulation, expansion is still observed due to the local expansion transmitted to the SL. N=30 independent simulations per condition, error: standard deviation.

Supplemental Movie captions

Movie S1. Related to Figure 2, 3 and 6

Deep layer intercalation and expansion depends on C3aR, as shown by time-lapse microscopy using the ex vivo assay (see Figure 2D). Mosaic-labeled deep layers (membrane – green, nuclei – red) explanted and covered with an unlabeled superficial layer were imaged through the superficial layer. Manually tracked cell trajectories demonstrate expansion (magenta tracks) in the control (CoMo treated deep layer) case (top row), but not in the C3aRMo case. Deep cells from lower layers intercalate into the top layer (cyan tracks), whereas cells from the top layer disappear when moving into deeper layers (blue track). Note that images of both CoMo and C3aRMo explants contain the same number of cells and the dark areas show unlabeled DCs. Any apparent difference in number of cells is due to natural variation in the mosaic labeling. Right column shows the trajectories without the fluorescent images. Framerate 3 minutes per frame.

Movie S2. Related to Figure 3

Cellular Potts model simulation of chemotaxis-driven epiboly. Two independent simulations demonstrate radial intercalation and expansion with chemotaxis (top, Chem+) and the lack of epiboly in the inhibited case (bottom, Chem-). Blue bars mark the initial position of the colored region (superficial cells – red, deep cells – green). Cell coloring is for visualization purposes only. Framerate 3 minute per frame (1800 MCS per frame).

Movie S3. Related to Figure 4

In vitro chemotaxis assay of deep and superficial layer explants. Deep and superficial layers explants are not motile when plated separately. When plated in proximity, control (CoMo) deep layer explants chemotax towards CoMo superficial layer explants. Chemotaxis is lost in experiments where the superficial layer is explanted from a C3Mo injected embryo, or when the deep layer is explanted from a C3aRMo injected embryo. Bottom row shows trajectories of 10 explants. Framerate 3 minutes per frame.

Movie S4. Related to Figure 4

Deep layer explants chemotax towards C3a source. Inverted bright-field time-lapse recordings of deep cell explants plated near the vicinity of C3a-soaked beads (circled). Explants do not chemotax towards beads soaked in C3aDesArg (left), a variant of C3a that is unable to bind to its receptor (C3aR). Bottom row shows trajectories of 10 explants. Framerate 3 minutes per frame.

Movie S5. Related to Figure 4

Apical protrusions (A, B) and protrusive activity (C, D) in deep layers covered with superficial layer in our ex vivo assay. Mosaic-labeled deep cells explanted and covered with an unlabeled superficial layer were imaged through the superficial layer. Control (CoMo) deep cells produce protrusions (A) and exhibit more protrusive activity (C, pseudo-colored to yellow) than C3aRMo treated deep cells. Framerate 3 minutes per frame.

Movie S6. Related to Figure 6

In vivo expansion of the blastocoel roof is dependent on C3aR. Animal pole of Xenopus embryos imaged from stage 8 show expansion in control (CoMo injected) case. This expansion is reduced when C3aR is inhibited (C3aRMo). Left column shows fluorescent time-lapse (membrane – green, nuclei – red) movie with manual trajectory overlay. Right column shows the trajectories only. Framerate 10 minutes per frame.

Movie S7. Related to Figure 7

Model simulation of epiboly at altered chemoattractant levels. Two independent simulations showing epiboly with 0.5x (top) or 1.5x (bottom) levels of chemoattractant compared to simulations shown in Movie S2. Both expansion and thinning is reduced when chemoattractant levels are halved, and increased with increased chemoattractant levels. Colors and marking as in Movie S2. Framerate 3 minute per frame (1800 MCS per frame).

Supplemental Experimental Procedures

Morpholinos and peptides

Xenopus C3a and C3aDesArg peptides were synthesized as previously described (Carmona-Fontaine et al., 2011). The following Morpholino oligomers were designed according to recommendations of Gene Tools or using published data:

- C3Mo: 5'-ACTGGACAATGTGCAAACTTTGAAT-3';
- C3aRMo and CoMo: as published (Carmona-Fontaine et al., 2011);
- E-cadherin Mo: as published (Nandadasa et al., 2009).

The effectiveness of the C3Mo is demonstrated in Figure S2F and S2G using western blot. The fibronectin antibody used was the mAb 4H2 previously characterized in the Xenopus system (Ramos and DeSimone, 1996).

Staining and Imaging

Whole-mount in situ hybridization was performed as described previously (Harland, 1991).
For in vivo time-lapse imaging of the SL, the animal pole of embryos was co-injected with nuclear RFP and membrane GFP constructs at the 8-cell stage, together with either CoMo or C3aRMo constructs. Embryos were imaged from the animal pole using fluorescence confocal microscopy (Leica, Upright-SP8) with several optical sections that were later max-projected using ImageJ. In vitro imaging was carried out using standard time-lapse fluorescence microscopy. Images were captured every 3 or 10 minutes as indicated.
Automatic tracking of cell nuclei of in vivo SL (Figure 6E and 6F) was performed using the DCU CIPA TRACKER software (Hossain et al., 2011; Thirusittampalam et al., 2013). Manual tracking was done using ImageJ's Manual Tracker plugin.
Adhesion and sorting assays were performed as previously described (Carmona-Fontaine et al., 2011).

Chemotaxis assay

For in vitro chemotaxis deep and superficial layers of the BCR were excised from embryos between stages 10 to 12 and separated by shaving the deep layer from the superficial in 3/8 NAM. Explants were plated on FN-coated (10µg/mL, Sigma) plastic dishes filled with DFA (Keller, 1978). Chemotaxis assays were performed and analyzed as described previously (Carmona-Fontaine et al., 2011). Briefly, explants of deep and superficial layers from stage 10 embryos were placed either separately, or at a distance of approximately one explant width. A similar procedure was carried out for C3a-coated and C3aDesArg-coated beads. A minimum of 10 explants were imaged for at least 6h for each condition. Chemotaxis indices were calculated using the Chemotaxis and Migration Tool for ImageJ (Ibidi).

Ex vivo tissue explant

Ex vivo intercalation assays were performed as previously described (Marsden and DeSimone, 2011). Briefly, embryos were injected to label the membranes and nuclei of cells in a mosaic fashion. The DL was explanted as described above and covered with SL from unlabeled embryos. The whole explant was covered with a glass cover slip to prevent curling of the SL. The optical penetration depth was limited to the top DC layer, allowing the study of apical DC activity and quenching all signals from the deeper regions. Membrane protrusion activity was determined by thresholding the level of membrane GFP staining signal. A higher signal indicates more membrane overlap caused by the wrinkling, or ruffling, of the cell membranes. The threshold was set to levels corresponding to a smooth cell membrane. Areas with a signal above the threshold were considered as areas of protrusive activity. Intercalation percentages for Figure 2F were calculated as the number of fluorescent cells appearing / disappearing over the time-lapse recordings in relation to the total number of fluorescent cells in the last frame of the recordings.

Computer simulations

To model our hypothesis we chose the cellular Potts model (CPM) framework (Graner and Glazier, 1992; Glazier and Graner, 1993; Szabó and Merks, 2013), a stochastic Monte-Carlo based model system. Cells are represented as connected domains on a grid with an integer, *σ(x)*, denoting the cell id at every grid point *x*. Cell-free areas are designated with a zero id. The same grid is used to describe the concentration field with *c(x)* at every point *x*. Each configuration *ξ* is characterized with a scalar valued function, the Hamiltonian:

$$H(\xi) = \sum_{i=1}^{N} \lambda_V (V(i) - V_T(i))^2 + \sum_{<x,y>} J_{x,y}\big(1 - \delta(x,y)\big) \tag{1}$$

The first sum runs through the cells and contains the deviation of cell volume *V(i)* from a designated target volume $V_T(i)$ scaled with a model parameter λ_V. The second term sums cell boundaries with weights $J_{x,y}$ corresponding to boundaries between cell types *x* and *y*. The δ(x,y) is the Kronecher-delta function: *δ(x,y)=1* if *x=y*, otherwise *δ(x,y)=0*. Cell movement is the result of a series of attempts to update cell ids on the grid. In an attempt a grid point, *a*, and one of its neighbors, *b*, are selected at random, and the id from site *b*, *σ(b)*, is copied into site *a*. This attempt is then evaluated and accepted with a probability:

$$P(\sigma(b) \to a) = \min(1, \exp\{w(\sigma(b) \to a) - \Delta H(\sigma(b) \to a)\}). \tag{2}$$

The function *ΔH*(*σ(b)*→ *a*) is the change in the Hamiltonian resulting from the copy attempt, while *Δw*(*σ(b)*→ *a*) is defined as:

$$w(\sigma(b) \to a) = \lambda_c\big(C(b) - C(a)\big) + \sum_{i=\{\sigma(a),\sigma(b)\}} \Delta r_i \cdot \sum_{j\epsilon\{n(i)\}} \lambda_{i,j} \frac{r_{i,j}}{|r_{i,j}|} \cdot \big(|r_{i,j}| - d_{i,j}\big), \tag{3}$$

with *C(a)* describing the saturated concentration at point *a* with saturation parameter *s* as

$$C(a) = \frac{c(a)}{s \cdot c(a) + 1}. \tag{4}$$

These rules give rise to cells with a volume fluctuating around a given value (volume term, Eq. 1), a surface tension-like cell adhesion (adhesion term, Eq. 2). Chemotaxis is implemented in the usual way by biasing cell displacements towards higher concentrations of the *c(x)* field (chemotaxis term, Eqs. 3 and 4). Cells are allowed to attach to one another upon certain conditions through a conditional attachment term (elastic term, Eq. 3) with Δr_i denoting the displacement of cell *i*'s center of mass, n_i the connected neighbors of *i*, $r_{i,j}$ the vectorial distance of cells *i* and *j*, and $d_{i,j}$ the target distance of connected cells *i* and *j*. This elastic term allows the construction of the epithelium and adhesion between the deep and superficial layers by forming elastic connections between cells as described below. The unit of time in the simulations is the Monte Carlo step (MCS) which is N copy attempts where N is the number of grid points in the system.

The chemoattractant, C3a, is diffusing and decaying with a constant rate:

$$\partial_t c(t,x) = D\nabla^2 c(t,x) - d_c \cdot c(t,x) \tag{5}$$

and is produced by the cells such that $c(x)=c_0$ = constant for every point *x* within the secreting cells. Concentrations at the top and bottom boundaries are set to zero, as any chemoattractant is expected to be diluted infinitely within the blastocoel cavity or outside the embryo. Concentrations at the simulation's side

boundaries are constructed to prevent gradients forming along the layers horizontally. This is achieved by copying the concentration values at 30 sites from the side edge to all the sites up to the edge:

$$c_L(x) = c_L(i,j) = c(A,j) \tag{6}$$

for the left edge of the simulation, where *A=30* demarks the outside margin in the simulation. Similarly on the right edge:

$$c_R(x) = c_R(i,j) = c(W-A,j) \tag{7}$$

where *W=400* is the width of the simulation field. In both cases *j* runs through the complete height, 80 lattice sites, of the simulation area.

Cell configurations in the simulations represent a single-cell thick quasi-2D cross sectional slice of the blastocoel roof, including two layers of deep cells (DCs) and a single layer of superficial cells (SCs, see Figure 3B). In simulations exploring the effect of DC numbers on epiboly (Figure S7), simulations are initiated with 6 layers of DCs and a given percentage of the DC population is then selected and removed at random. The SL and DL are separated by a thin and rigid layer of “frozen cells” (“wall cells” or WCs) that serve to prevent DC intercalation into the superficial layer (SL), and to prohibit DCs from exerting unrealistic pressure on the SL. Since cell movement in the model is relative to the simulation grid, DCs could easily push the SL further out as if they were plated on a 2D substrate. However, in the embryo no such substrate exists as the cells adhere only to each other, and such a pressure would not be feasible. WCs are minimal in size (a single lattice site), and are not allowed to move, and form the wall layer (WL).

The epithelium is constructed as a chain of cells attached to the WCs. If the contact area of a SC and the WL is less than a threshold of 2 lattice sites, the SC is forced towards the WL by applying a constant force on the center of mass of the SC. Similarly, if the contact area of two neighboring SCs falls below a threshold of 2 units, the two cells are forced to move towards each other by the means of the elastic constraint (Eq. 3). Otherwise the links among SCs remain inert (by setting $\lambda_{i,j} = 0$). Each SC is connected to exactly two other SC neighbors via such “epithelial” connections. To allow full epithelial integrity, SCs are also required to remain simply connected (via the Global Connectivity plugin; Swat et al., 2012). This construction of the epithelium allows the layer to adjust its height and length without enforcing a fixed cell-cell distance, but still keeps the integrity of an epithelium.

DCs undergo chemotaxis towards the chemoattractant (Eq. 3) and therefore towards the SL. When in contact with the wall cell layer (WL), an elastic link between the cell centers of the DC and the SC immediately above the center of mass of the DC is established with a fixed probability (0.999), a mechanism similar to what has been proposed for keratinocytes (Czirók et al., 2013). Such links are cut whenever a DC loses contact with the WL, or the link reaches twice its original length, or with a small constant probability ($3x10^{-4}$). These rules allow the relative sliding of the deep and superficial layers.

Each SC and DC maintain an individual internal cell-cycle clock that is initiated at random and makes sure that each such cell undergoes exactly one cell division during the course of the simulations. The number of divisions is based on a previous study (Keller, 1980) and our current observation of in vivo time-lapse recordings that show that SCs divide approximately once or twice in the two-dimensional plane during epiboly. Assuming that there is no privileged orientation of divisions within the plane of the tissues (Longo et al., 2004, Woolner and Papalopulu, 2012), cells should divide once in the plane of our quasi-2D setup and once perpendicular to it. The latter would be unnoticed in our simulations; therefore cells in the model divide only once. Mitotic spindle orientation in SCs is always parallel to the plane of the SL following experimental observations (Longo et al., 2004, Woolner and Papalopulu, 2012). In our model DCs divide along their minor cell axis. Before cell division all connections are removed and automatically regenerated

following the same rules as above. In case of epithelial links (SC-SC links), the daughter cells are connected to each other and to one of the former connection partner of the parent nearest to them.

To achieve neutral boundary conditions that allow free expansion of the tissue but still prevent it from collapsing into a compact cluster, anchor points were introduced near the simulation sides above the WL. Anchor points, similar to WCs, are fixed (frozen) cells with a minimal size. SCs touching an anchor point form an "epithelial" connection with it, as described above. SCs that have their center of mass outside the anchor points and are not connected to an anchor point are considered outside the simulation area. Additionally, if all epithelial neighbors of a SC are connected to an anchor point, it is also considered to be outside. SCs outside the simulation are disconnected from all other cells and lose their volume constraint (but cannot expand over their original size). Similarly, deep cells that are outside the anchor points and are not connected to any SCs, lose their volume constraint, their ability to connect to any cell, and their ability to chemotax. Since outside cells lack volume constraint, they are free to disappear under pressure, but cannot re-enter the simulation (Voss-Böhme, 2012). In case no DCs are present under the anchor point in a 5x5 lattice site area, a new DC is introduced at this position. Anchor points are set at 30 lattice sites distance from the sides of the total simulation arena width of 400 lattice sites.

As expansion occurs by displacement of the whole tissue, mechanical equilibrium is required to prevent artificial wrinkles produced by a low speed of sound in the model (Szabó et al., 2012). This effect is overcome by introducing sufficiently high time resolution (Szabó et al., 2012). Therefore, we simulate 1 minute of real time using 600 computational time steps (Monte Carlo steps, MCS). Spatial calibration is based on relating the average cell size of 40µm to the simulated cell size of 8 lattice sites resulting in 5µm / pixel resolution. Using this calibration, the diffusion coefficient of the chemoattractant is set to 0.68, relating to $1.7x10^{-10}$ m^2/s measured for Sdf-1 in cell culture medium (Lin and Butcher, 2006), and the decay rate is set to $4x10^{-5}$, relating approximately to a typical 1h protein half-life (Belle et al., 2006). For simulations of the in vitro chemotaxis assay a time calibration of 1 min = 10MCS was used, as in this experimental assay the cells are attached to the underlying substrate and the culture is equilibrated quicker. For these simulations parameters were scaled accordingly to keep the same real values, therefore diffusion parameter is set to 40 and decay rate is set to $2.4x10^{-3}$, and a simulation area of 400x400 lattice sites was used with two clusters of 23 cells initialized with their centers 100 lattice sites apart, giving rise to an approximate 50 lattice site gap between the clusters. Adhesion parameters in all simulations were set to provide adhesive SCs: $J_{SC,SC} = 2$, $J_{SC,medium} = 3$; slightly adhesive DCs: $J_{DC,DC} = 1.8$, $J_{DC,medium} = 1$; adhesive WCs: $J_{WC,DC} = J_{WC,SC} = 1$. Initial cell volumes are set to 64 lattice sites, chemotaxis saturation coefficient is set to $s=0.1$ (Eq. 4). The relative importance of the probability terms are set to: $\lambda_V=1$, $\lambda_c=100$, $\lambda_{i,j}=1$ for SC-DC connections and $\lambda_{i,j}=50$ for SC-SC connections.

Supplemental References

Belle, A., Tanay, A., Bitincka, L., Shamir, R., and O'Shea, E.K. (2006). Quantification of protein half-lives in the budding yeast proteome. Proc. Natl. Acad. Sci. 103, 13004–13009.

Glazier, J.A., and Graner, F. (1993). Simulation of the differential adhesion driven rearrangement of biological cells. Phys. Rev. E 47, 2128–2154.

Harland, R.M. (1991). Xenopus laevis: Practical Uses in Cell and Molecular Biology (Elsevier).

Hossain, M.J., Whelan, P.F., Czirók, A., and Ghita, O. (2011). An active particle-based tracking framework for 2D and 3D time-lapse microscopy images. Conf. Proc. ... Annu. Int. Conf. IEEE Eng. Med. Biol. Soc. IEEE Eng. Med. Biol. Soc. Annu. Conf. 2011, 6613–6618.

Lin, F., and Butcher, E.C. (2006). T-cell chemotaxis in a simple microfluidic device. Lab Chip 6, 1462.

Nandadasa, S., Tao, Q., Menon, N.R., Heasman, J., and Wylie, C. (2009). N- and E-cadherins in Xenopus are specifically required in the neural and non-neural ectoderm, respectively, for F-actin assembly and morphogenetic movements. Development 136, 1327–1338.

Ramos, J.W., and DeSimone, D.W. (1996). Xenopus embryonic cell adhesion to fibronectin: position-specific activation of RGD/synergy site-dependent migratory behavior at gastrulation. J. Cell Biol. 134, 227–240.

Szabó, A., and Merks, R.M.H. (2013). Cellular potts modeling of tumor growth, tumor invasion, and tumor evolution. Front. Oncol. 3, 87.

Thirusittampalam, K., Hossain, M.J., Ghita, O., and Whelan, P.F. (2013). A Novel Framework for Cellular Tracking and Mitosis Detection in Dense Phase Contrast Microscopy Images. IEEE J. Biomed. Heal. Informatics 17, 642–653.

Voss-Böhme, A. (2012). Multi-Scale Modeling in Morphogenesis: A Critical Analysis of the Cellular Potts Model. PLoS One 7, e42852.
